# Juggling cadmium detoxification and zinc homeostasis: A division of labour between the two *C. elegans* metallothioneins

**DOI:** 10.1016/j.chemosphere.2023.141021

**Published:** 2024-02

**Authors:** Yona J. Essig, Oksana I. Leszczyszyn, Norah Almutairi, Alexandra Harrison-Smith, Alix Blease, Sukaina Zeitoun-Ghandour, Sam M. Webb, Claudia A. Blindauer, Stephen R. Stürzenbaum

**Affiliations:** a Analytical, Environmental and Forensic Sciences Department, King's College London, London, UK; b Department of Chemistry, University of Warwick, Coventry, Gibbet Hill, UK; c Stanford Synchrotron Radiation Lightsource, SLAC National Accelerator Laboratory, 2575 Sand Hill Road, Menlo Park, CA, 94025, USA

**Keywords:** Cadmium, Detoxification, Metallothionein, *Caenorhabditis elegans*

## Abstract

The chemical properties of toxic cadmium and essential zinc are very similar, and organisms require intricate mechanisms that drive selective handling of metals. Previously regarded as unspecific “metal sponges”, metallothioneins (MTLs) are emerging as metal selectivity filters. By utilizing *C. elegans mtl-1* and *mtl-2* knockout strains, metal accumulation in single worms, single copy fluorescent-tagged transgenes, isoform specific qPCR and lifespan studies it was possible to demonstrate that the handling of cadmium and zinc by the two *C. elegans* metallothioneins differs fundamentally: the MTL-2 protein can handle both zinc and cadmium, but when it becomes unavailable, either via a knockout or by elevated cadmium exposure, MTL-1 takes over zinc handling, leaving MTL-2 to sequester cadmium. This division of labour is reflected in the folding behaviour of the proteins: MTL-1 folded well in presence of zinc but not cadmium, the reverse was the case for MTL-2. These differences are in part mediated by a zinc-specific mononuclear His_3_Cys site in the C-terminal insertion of MTL-1; its removal affected the entire C-terminal domain and may shift its metal selectivity towards zinc. Overall, we uncover how metallothionein isoform-specific responses and protein properties allow *C. elegans* to differentiate between toxic cadmium and essential zinc.

## Highlights

• Cd and Zn was quantified in single nematodes.
• Single copy integrated metallothioneins in wild-type and mutant nematodes.
• *Mtl-1* expression is low but rapidly induced upon metal exposure.
• *Mtl-2* is constitutively expressed (at high levels) in the gut cells.
• MTL-1 and MTL-2 proteins are characterized by unique folding behaviours.

## Graphical abstract

**Figure:**
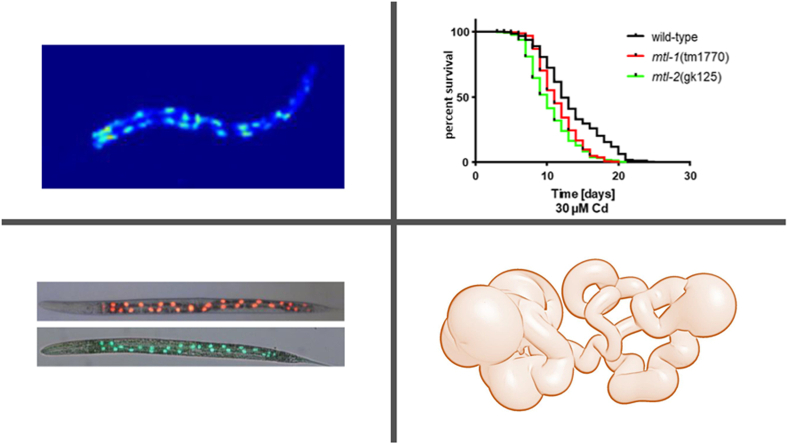

## 1 Introduction

The examination of metal toxicity is important because of the scale of global industrialisation and the consequential increase in metal demand (Gordon et al., 2006), which in turn triggers environmental risks. Some metal ions are important to organisms as they play a crucial role in many biochemical reactions (Fairweathertait, 1983). They are often involved in catalytic processes and/or stabilise tertiary or quaternary structures in proteins (Halliwell and Gutteridge, 1990). Zinc, for example, is the second most abundant trace metal in the human body and an essential nutrient in all organisms. Approximately 3000 human proteins utilise zinc as a cofactor (Maret, 2011), with involvement in DNA synthesis, gene expression, enzymatic catalysis and apoptosis (John et al., 2010). A tight homeostatic control of zinc is therefore crucial as any imbalance (dearth or excess) can lead to disease progression.

Other metals are non-essential with no (known) beneficial function to biological systems. Cadmium, for example, is included on the “Top 20 Hazardous Substances Priority List” published by the Agency for Toxic Substances and Disease Registry/Environmental Protection Agency (ATSDR), mainly due to its toxicity and carcinogenicity. The cadmium carbonic anhydrase in marine diatoms is, to our knowledge, the only example where an organism utilises cadmium as a catalytic metal atom in a zinc-depleted environment, such as the ocean (Xu et al., 2008). Cadmium is frequently used by modern industries involved in battery and solar cell production, electroplating and silver soldering industries as well as plastic and paint manufacturing. Cadmium is also often released into the environment as a by-product of metal smelting, refining and mining (Carruthers and Smith, 1979; Genchi et al., 2020). It is therefore of paramount importance that organisms develop pathways that guard cells from toxic accumulation of Cd.

Zn^2+^ and Cd^2+^ are the only biologically relevant ionic forms and they are physiochemically similar, thus can compete for the same binding sites within proteins (Brzoska and Moniuszko-Jakoniuk, 2001). The substitution of the essential cofactor Zn^2+^ with a toxic Cd^2+^ ion can influence biochemical processes of metalloenzymes (Jacobson and Turner, 1980). However, Cd^2+^ not only interferes with Zn^2+^ homeostasis (Earley et al., 2021) but can also impact Ca^2+^ physiology, causing cellular damage, protein denaturation, indirect generation of reactive oxygen species (ROS) and DNA strand break (Branca et al., 2022; Lopez et al., 2006; Hengstler et al., 2003; Youngs et al., 2000). These phenomena explain why an occupational or environmental exposure to high concentrations of cadmium increases the risk of many diseases, including lung, breast, urinary bladder, pancreas, nasopharynx and prostate cancer (Huff et al., 2007; Mężyńska and Brzóska, 2018). In contrast, zinc supplementation has been shown to buffer the toxic effects of cadmium (Boughammoura et al., 2021).

Metal detoxification and metal homoeostasis are intertwined with the metallothionein status in metal-exposed organisms (Klaassen et al., 1999; Kägi and Schaffer, 1988; Kägi, 1991; Kojima and Hunziker, 1991; Sutherland et al., 2010; Krężel A and Maret, 2021; Norberg and Norberg, 2022). Metallothioneins (MTs), discovered in 1957 (Margoshes and Vallee, 1957), are small cysteine-rich proteins with no or few aromatic amino acids. Under normal physiological conditions Cu^+^ and/or Zn^2+^ bind to MTs predominantly in cysteine (Cys) sulfur-based metal-thiolate clusters (Freisinger and Vašák, 2013). Typically, owing to the soft character of thiolate, the borderline Zn^2+^ ion can be readily replaced by the much softer Cd^2+^, if the latter is presented to the protein. *Homo sapiens* has 4 major subfamilies of MTs (MT1 – MT4) with MT1 being the most intricate, consisting of 18 isoforms. In contrast, the less complex nematode *C. elegans* has only two MTs (named, in accordance with the established three-letter worm nomenclature, MTL-1 and MTL-2 (“MeTaLlothionein”) (Kugawa et al., 1994), and their expression is induced by heat shock (Freedman et al., 1993), oxidative stress (Zeitoun-Ghandour et al., 2011; Hall et al.) and upon heavy metal challenge, in particular cadmium, but also zinc.

The two *C. elegans* MTs differ in four main ways. Firstly, the sequence length: MTL-1 possesses a 15-aa insertion at the C-terminus (notably including 2 histidines and a cysteine) and MTL-2 has a 3-aa insertion in the N-terminal half (Stürzenbaum, 2009). Secondly, the metal binding capacity: MTL-1 can bind 7 metals but MTL-2 only 6 metals (Bofill et al., 2009; Zeitoun-Ghandour et al., 2010; Leszczyszyn et al., 2010). Thirdly, metal-binding affinity: MTL-1 and MTL-2 have similar Zn^2+^ binding affinity, but the average Cd^2+^ binding affinity of MTL-2 is almost two orders of magnitude higher than that of MTL-1. Finally, differential expression: previous reports have suggested that only *mtl-1* is constitutively expressed in cells of the lower pharyngeal bulb, whereas *mtl-1* and *mtl-2* expression are strongly induced in the intestinal cells following an exposure to cadmium or zinc (Freedman et al., 1993; Dietrich et al., 2016; Cui et al., 2007; Swain et al., 2004). A recent report demonstrated that *mtl-1* (but not *mtl-2*) expression was significantly induced in zinc and manganese co-exposed *C. elegans* (Baesler et al., 2021).

Given the importance of essential metal homeostasis and toxic metal elimination, surprisingly little is known about the interplay between the two MT isoforms, and how their protein structures may relate to biological function(s); a shortfall this paper aims to explore in detail.

## 2 Materials and methods

### 2.1 Strains

*C. elegans* wild-type Bristol N2 and *mtl-2* (gk125) were obtained from the *Caenorhabditis* Genetics Centre (University of Minnesota, Minneapolis, MN, USA) and *mtl-1* (tm1770) from the National Bioresource Project Core Facility (Mitani Laboratory, Tokyo Women's Medical University School of Medicine). The P*mtl-1*:GFP and P*mtl-2*:mCherry were generated by MosSCI single copy integration in collaboration with KNUDRA Transgenics, USA. The P*mtl-1*:GFP;*mtl-2* (gk125) and the P*mtll-2*:mCherry;*mtl*-*1* (tm1770) strains were created in our laboratory. All stains were backcrossed 4 times and genotyped by nested PCR.

### 2.2 Metal sample preparation

Zinc (ZnCl_2_, ultra dry, purity 99.999%) and cadmium (CdCl_2_, 99.99+%) were dissolved in double filtered, double autoclaved HPLC water and added in equimolar concentrations to nematode growth media (NGM) and *E. coli* OP50. All solutions for biophysical characterisation of purified proteins were prepared in degassed Milli-Q water. Unless stated otherwise, chemicals were purchased from Sigma-Aldrich. Nematodes were grown in Petri dishes on NGM (nematode growth media) and fed, ad libitum, *E. coli* OP50 and exposed to inoculated NGM plates from age synchronised L1 larval stage to L4 stage (48 h).

### 2.3 X-ray fluorescence imaging (XFI)

The accumulation of zinc in the body of a single nematode was visualised by X-ray fluorescence imaging (XFI) conducted at the Stanford Synchrotron Radiation Lightsource (SLAC). Details of the method were described previously (Essig et al., 2016). Briefly, XFI was performed on single age-matched (synchronised) nematodes (L4) at incident energy 10 keV, selected with a Si(111) double crystal monochromator. The X-ray beam was focused to a 2 × 2 μm spot size using a Rh coated Kirkpatrick-Baez (KB) mirror pair (Xradia Inc (now Zeiss), Pleasanton, CA, USA) equipped with Xspress3 electronics (Quantum Detectors); the intensity of the fluorescence lines of the elements of interest from the sample was monitored. The obtained images (3 biological replicates per condition) were analysed using the Micro Analysis Toolkit (Webb, 2011).

### 2.4 Laser ablation-inductively coupled plasma-mass spectrometry (LA-ICP-MS)

The accumulation of cadmium was quantified in individual nematodes by laser ablation-inductively coupled plasma-mass spectrometry (LA-ICP-MS) (Teledyne Photon Machines Inc.; Thermo Fisher Scientific). Synchronised L1 worms were collected at L4 stage and washed multiple times with M9 buffer, then washed twice rapidly with ice cold methanol. The worms (10 replicates/condition) were dropped onto a glass slide and left to dry at room temperature for subsequent metal screening and imaging. Each worm was scanned with a spatial resolution of 5 μm using the same settings to facilitate visual comparison between conditions defined as the average of pixel intensity within a single worm. ICP-MS and positional data were reconstructed to generate elemental images using the HDF-based Image Processing software (HDIP, Teledyne Photon Machines Inc.). A bespoke pipeline, written in Python coding language (version 3.8), was used for image analysis and statistics on reconstructed data generated by HDIP, as well as Matplotlib modules in Python (Hunter, 2007) to generate heat maps.

### 2.5 Fluorescence microscopy of transgenic *C. elegans*

Fluorescence images were taken using a Nikon inverted fluorescence microscopy camera (Nikon TE2000-S) or a confocal microscope (Nikon A1R).

### 2.6 Quantitative real-time PCR

Extraction of total RNA was performed on approximately 5000 synchronised L4 nematodes, using the Zymo Quick-RNA™ kit (Cambridge Bioscience) as recommended by the manufacturer, however including an initial vortexing step (3 min) with acid-washed glass beads (Sigma-Aldrich). The total RNA was analysed spectrophotometrically (NanoDrop® ND 1000 Spectrophotometer) and separated on a 1% agarose gel to assess the quantity and quality, respectively. cDNA was synthesised with 1000 ng of RNA using an oligo dT primer (5’–(T)_20_ VN–3′) and M-MLV reverse transcriptase (Promega). Quantitative PCR of *mtl-1, mtl-2* and *rla-1* was performed using the ABI Prism 7500 Fast System platform (Applied BioSystems). Primers were designed to be exon-spanning and probes were sourced from the Universal ProbeLibrary (Roche). For each qPCR reaction a master-mix was prepared containing 5 μL ROX-Buffer (Roche), 0.1 μL probe, 1 μL primers (10 pM) and made up with H_2_O to 8.8 μL total volume. The cDNA template was diluted ten-fold and 2 μL was added. qPCR was performed using the standard ABI Prism cycling conditions (2 min at 50 °C, 10 min at 95 °C, 40 cycles, 15 sec 95°C and 1 min 60 °C). Data were analysed via the ABI 7500 Fast Software v2.3 and expression fold change determined by the 2^˗ΔΔCt^ method. To determine the copy number, the respective amplicons were cloned into a pGEM®T-vector and a serial dilution of the cloned product was used to determine the corresponding CT-values by qPCR. The CT-values were plotted against the initial plasmid concentration and the linear equation was calculated using the linear regression standards (Fig. S16), which allowed the gene concentration to be determined at any CT-value. The copy number was calculated via the molecular mass of the plasmid with the ligated gene, the number of molecules per mole and the Avogadro constant. The copy number refers to the total number of the gene of interest in each sample and was expressed as the total copy number of *mtl-1* or *mtl-2* per *rla-1*.

### 2.7 Life span

The life span assay involved the monitoring of 400 individual nematodes (per strain/condition) from L1 until death. To prevent overcrowding, worms were transferred daily to freshly prepared plates until the egg laying process ceased. The survival was determined by tapping the plate or stroking the nematode with a platinum wire. Censored and dead nematodes were counted each day. The data were analysed and processed by means of the Kaplan-Meier method.

### 2.8 Protein expression and purification

Cloning and production of constructs for wild-type MTL-1 and MTL-2 in pET29a, using cloning sites Sal 1 and Nde 1, and details of protein purification are reported in (Zeitoun-Ghandour et al., 2010). Briefly, all proteins were expressed in *E. coli* Rosetta™2 (DE3)pLysS (Merck) without any fusion tags, using 50 mg L^−1^ kanamycin and 34 mg L^−1^ chloramphenicol as selective antibiotics. Expression was induced with 0.5 mM IPTG, with simultaneous addition of either 0.5 mM ZnSO_4_ or CdCl_2_. Purification was achieved by chemical fractionation (Vasák, 1991) followed by size-exclusion chromatography (Superdex G25, GE Healthcare). For most samples, the latter was carried out in 20 mM NH_4_HCO_3_ buffer (pH 7.8), facilitating subsequent ESI-MS analysis. When required, buffer was exchanged by gel filtration (Sephadex G-25, GE Healthcare PD-10 columns), and samples were concentrated using Amicon Ultra centrifugation filters (MWCO 3 kDa). ^15^N or ^13^C/^15^N-labelled proteins for NMR spectroscopy were expressed in M9 media containing ^15^NH_4_Cl or ^13^C-glucose/^15^NH_4_Cl (CK Isotopes) as sole sources of nitrogen and carbon. Metal supplementation and induction were the same as for unlabelled proteins. Samples for ^111^Cd NMR spectroscopy were produced by removing bound metal (Zn^2+^ for MTL-1 and Cd^2+^ for MTL-2) at low pH (pH 1), followed by gel filtration at pH 2 (Sephadex G-25, GE Healthcare PD-10 columns) under an inert atmosphere, addition of a slight excess of ^111^CdCl_2_ prepared from ^111^CdO (99% ^111^Cd, CK Isotopes), and subsequent adjustment to the desired pH using 1 M deuterated Tris base (Tris-D_11_; 98% D, CK Isotopes).

### 2.9 Construction of mutant proteins

The structural importance of the C-terminal insert was studied by generating an *mtl-1* construct with a shortened C-terminus to resemble *mtl-2* and an *mtl-2* construct with an extended C-terminus corresponding to that of native *mtl-1*. In detail, the PCR products of wild-type (native) *mtl-1* and *mtl-2* were utilised as templates to amplify *mtl-1* and *mtl-2* fragments with the addition or deletion of the C-terminal sequence respectively.

The purified PCR products were cloned into (−) *S*-tag pET29a and confirmed by sequencing (GATC Biotech, Germany). The MTL-2+MTL-1_57–71 tail mutant was further modified by Quikchange site-directed mutagenesis (Agilent), mutating E57 to histidine.

### 2.10 Non-denaturing electrospray mass spectrometry

25 μM protein samples were prepared in 10 mM ammonium acetate, pH 7.4. Methanol (10% v/v final concentration) was added to the protein samples immediately prior to infusion via a syringe pump at a rate of 250 mL h^−1^. Positive-ion electrospray mass spectra of the mutant proteins (25 mM, 10 mM ammonium acetate, pH 7.4, 10% methanol) were acquired on either a Bruker Daltonics HCTultra Ion Trap or a Bruker Daltonics MicrOTOF mass spectrometer. Typical acquisition times were 1.5 min using an *m*/*z* range of 500–3000. Data were averaged, smoothed, baseline-subtracted and deconvoluted onto a true mass scale in Bruker Daltonics Compass DataAnalysis.

### 2.11 Zinc and cadmium affinity measurements by ^19^F NMR spectroscopy

Affinity measurements were carried out using the method outlined in (Hall et al.). Briefly, samples of Cd- and Zn-loaded MTL mutants (MTL-1Δ57-71 and MTL-2+MTL-1_57–71), approximately 480 μM with respect to metal ion concentration, were prepared in 10 mM Tris-Cl (pH 8.1, 10% D_2_O). Accurate metal concentrations were determined using Inductively Coupled Plasma-Optical Emission Spectroscopy (ICP-OES, PerkinElmer Optima 5300 DV). 5F-BAPTA (1,2-bis(2-amino-5-fluorophenoxy)ethane-N,N,N′,N′-tetraacetic acid, Molecular Probes Inc.; 4 mM final concentration) was added to each sample, followed by incubation over night at room temperature. Direct observe 1D^19^F NMR spectroscopy was carried out on a DRX400 spectrometer (Bruker) fitted with a QNP probe-head operating at 375.91 MHz for ^19^F. Chemical shifts are reported in relation to the signal of CCl_3_F at 0 ppm. Spectra were acquired at 298 K with a spectral width of 50 ppm, an acquisition time of 3.48 s and relaxation delay of 1.0 s, with 12k scans. FIDs were apodised with squared sine-bell functions and Fourier-transformed with 65k complex data points and baseline corrected. Spectra were processed with TOPSPIN v.2.1 software (Bruker). Calculations of apparent stability constants, valid for pH 8.1 and an ionic strength of 4 mM, were carried out using a published procedure (Hasler et al., 2000) and are based on log *K*_Zn-BAPTA_ = 9.91 and log *K*_Cd-BAPTA_ = 11.75.

### 2.12 NMR spectroscopy for structure determination

NMR samples were prepared in either 20 mM NH_4_HCO_3_ or 20 mM Tris-D_11_ buffers (in each case with 10% D_2_O, at pH values between 6.8 and 7.3, and protein concentrations between 0.5 and 1 mM), with addition of 20–50 mM NaCl; individual sample conditions are given in Figure captions. All isotopically enriched materials (^15^NH_4_Cl, ^13^C-glucose, ^111^CdO and Tris-D_11_) were purchased from CK Isotopes.

Standard protein NMR data were acquired on a Bruker Avance 700 Ultrashield spectrometer equipped with a TCI cryoprobe, with the following operating frequencies: 700.234 MHz for ^1^H, 176.08 MHz for ^13^C, and 70.95 MHz for ^15^N. Typically, all 2D and 3D spectra were acquired with a spectral width of 13 ppm in the ^1^H dimension, using either the WATERGATE sequence or pulsed field gradients for suppression of the water signal. 2D [^1^H,^1^H] TOCSY (60 ms mixing time) and [^1^H,^1^H] NOESY spectra (mixing times between 100 and 150 ms) were typically acquired with 4k datapoints in F2 and 512 increments in F1 and processed with 2k x 2k datapoints in the frequency domains. 2D [^1^H,^15^N] HSQC spectra (99–137 ppm in the ^15^N dimension for backbone and sidechain amides, and 85–235 ppm for imidazole ^15^N-Hδ2 and -Hε1 couplings) were acquired with 2k x 512 datapoints, using a coupling constant of ^1^*J* (^1^H,^15^N) = 90 Hz or ^2^*J* (^1^H,^15^N) = 30 Hz, respectively. A 2D [^1^H,^13^C] HSQC spectrum was acquired with the same number of datapoints over a spectral range of 8–158 ppm. 3D [^1^H,^15^N,^1^H] TOCSY-HSQC and [^1^H,^15^N,^1^H] NOESY-HSQC were acquired with 2k x 90 × 200 datapoints over a spectral range of 100–136 ppm in the ^15^N dimension. [^1^H,^1^H,^15^N] HNHA and HNHB datasets were acquired with 2k x 128 × 40 datapoints over the same spectral ranges. [^1^H,^15^N,^13^C] HNCA and [^1^H,^15^N,^13^C] HN(CO)CA were acquired with 2k x 40 × 64 datapoints using the same spectral range for ^15^N, and 38–70 ppm for ^13^C.

1D proton-decoupled ^111^Cd and 2D [^1^H,^111^Cd] HSQC NMR spectra were acquired on a Bruker DRX500 spectrometer equipped with a BBO probehead, operating at 106.06 MHz for ^111^Cd and 500.13 MHz for ^1^H. Spectral ranges for the 2D experiments were 13 ppm for ^1^H and 100 ppm for ^111^Cd, with 2k x 196 datapoints. Separate experiments using ^3^*J* (^1^H,^111^Cd) coupling constants of 30, 45 and 60 Hz were recorded. Data were Fourier-transformed into 2k x 1k datapoints after apodisation using squared shifted sine-bell functions in both dimensions.

### 2.13 Structure calculations

Sequential assignment was carried out in Sparky v. 3.114. For both proteins, structure calculations were performed in CYANA 2.1. Distance restraints were generated from 2D NOESY and 3D NOESY-HSQC (MTL-1 only) crosspeaks using CALIBA; these were also used to generate stereospecific assignments of CHβ protons and backbone angle restraints using HABAS (Güntert et al., 1991).

Assignment for Cd_6_MTL-2 (Appendix Table S2) was based on 2D ^1^H TOCSY and NOESY data and followed standard procedures (Putignano et al., 2018). Residues Cys 5, Asp 6 and Ser 13 were not assigned, and the NH resonances for Thr18, Asp 25 and Gly 50 were not observed. Initial structure calculations used 200 random starting structures and only considered these ^1^H NMR data derived restraints (although we found it necessary to define upper distance limits of 8.62 Å (the largest distance between sulfur atoms in a Cd–S cluster) for sulfur-sulfur distances, despite using uncharged cysteines); for the best 20 conformers, this gave backbone r.m.s.d. values of 3.26 ± 1.02 for residues 3 to 30 (domain 1) and 1.41 ± 0.49 Å over residues 31–61 (domain 2) after several rounds of refinement. To these refined restraints, metal-sulfur (2.52 Å) and sulfur-sulfur (4.50 Å) distance restraints were added for experimentally determined metal-cysteine connectivities, with metal ions introduced via a modified residue library, and using deprotonated cysteine residues. Only 5 out of 12 Cd-Cys connectivities were unambiguously assignable from the set of ^1^H,^111^Cd HSQC spectra; further connectivities were inferred by inspecting the structures resulting from initial refinements in combination with ambiguous ^1^H,^111^Cd crosspeaks where appropriate. Final r.m.s.d. values were 2.62 ± 0.80 Å over residues 3 to 61, 2.54 ± 1.05 Å for domain 1 (residues 3 to 30), and 1.18 ± 0.26 Å for domain 2 (residues 31 to 61).

For structure calculations of Zn_7_MTL-1, sequential assignment was facilitated by 3D [^1^H,^15^N,^13^C] HNCA and HN(CO)CA experiments, together with 2D and 3D TOCSY and NOESY data (Table S3). Residues Ala 1, Cys 2, Ser22-Lys23, Gln 66, Gly69-Ala72 could not be assigned. In addition to the distance restraints from 2D NOESY and 3D NOESY-HSQC data, Φ dihedral angles derived from a HNHA experiment, and a limited number of ξ-1 angles from a HNHB experiment were also used in the structure calculations. During initial refinements, it became clear that the available restraints were insufficient to define the fold of domain 1; thus, subsequent refinements focused on the separate domain 2 (residues 26–69; the remaining five C-terminal residues also lacked structural information and were not included in the final models). In the data from the ^13^C,^15^N-labelled sample, we noted the presence of a second species that gave rise to additional peaks for residues Cys29-Cys38, Ala41-Lys44, Cys 54, 55 Ala, Gln 59, Cys60, Ala 73 and His74. Only the stronger set of peaks was considered for structure determination. Initial models did not involve any metal-ligand restraints (other than defining an upper distance limit of 8.12 Å between Cys sulfurs as described above for MTL-2, with considering the smaller size of the Zn^2+^ ion), giving after refinement a backbone r.m.s.d. of 1.94 ± 0.57 Å over residues 26–69, for the best 20 models from 200 random starting structures. Subsequently, metal-ligand and ligand-ligand distance restraints for the His_3_Cys site G were added (including 3.76 Å between imidazole nitrogens of His53, His65 and His67, and 3.95 Å between the sulfur of Cys60 and each of the three imidazole nitrogens), and further refinement gave a backbone r.m.s.d. of 1.46 ± 0.30 Å. These models also involved the incorporation of three further Zn^2+^ ions, with one ion connected to each of Cys43, 47 and 52, but no further restraints.

## 3 Results and discussion

### 3.1 The dynamics of cadmium and zinc accumulation are dependent on metallothionein status

**Fig. 1:**
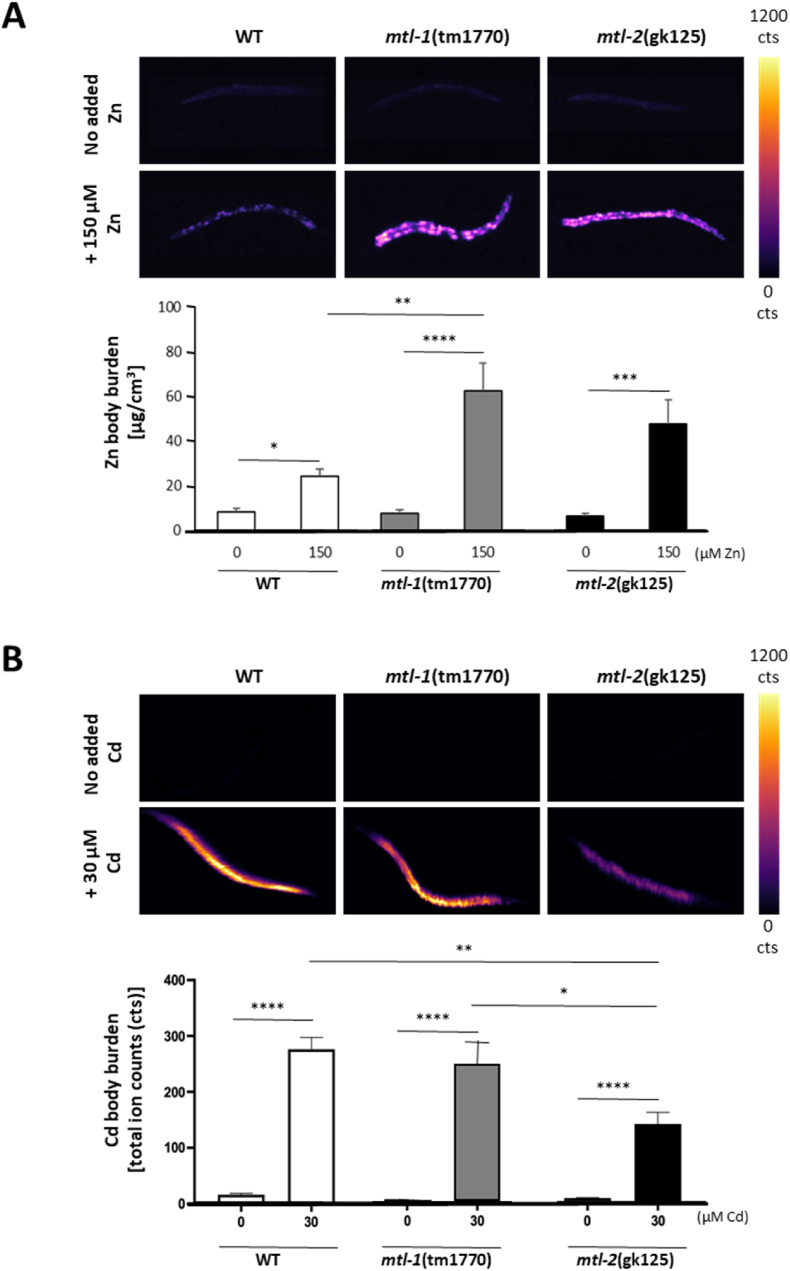
Metal accumulation in worms. Heat maps of individual wild-type and metallothionein mutant nematodes were generated (A) by X-ray fluorescence imaging (XFI) to quantify the accumulation of Zn following an exposure to 150 μM Zn or (B) by laser ablation-inductively coupled plasma-mass spectrometry (LA-ICP-MS) to determine the accumulation of Cd. Worms were raised on control plates or plates supplemented with either 150 μM zinc or 30 μM cadmium from L1 to L4 stage (for 48 h). Error bars represent mean ± SEM with n = 7 per condition. Statistical analysis was performed using one-way ANOVA. * denotes p ≤ 0.05, *** denotes p ≤ 0.001.

**Fig. 2:**
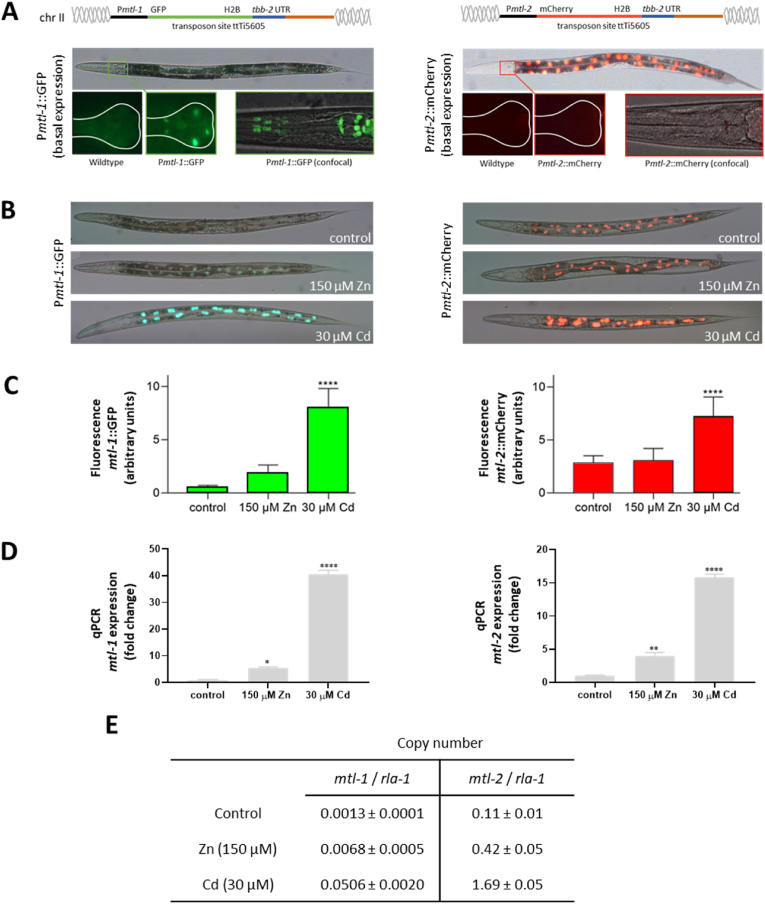
Transgenic nematodes P*mtl-1*:GFP and P*mtl-2*:mCherry generated by Mos1-mediated single copy insertion (MosSCI). (A). In the absence of metal-supplementation P*mtl-1*:GFP was expressed in five cells of the lower pharyngeal bulb and pharyngeal muscle cells. Conversely, P*mtl-2*:mCherry was observed in intestinal cells, but not the pharynx. (B) Challenging the two transgenic worms with either 150 μM Zn or 30 μM Cd revealed a strong increase in fluorescence in the intestinal cells. Images were captured at 20× magnification for the whole nematode body image and 100× magnification for the pharyngeal images. (C) Relative changes in fluorescence were quantified using age synchronous worms exposed from L1 to L4 (48 h), n = 10 per condition. (D) Isoform specific qPCR allowed the quantification of fold change expression of *mtl-1* and *mtl-2*. The relative induction potential was compared to unexposed control (mean ± SEM, normalised to *rla-1*) from 3 independent biological replicates). (E) The absolute copy number of *mtl-1* and *mtl-2* (normalised to *rla-1*) was also determined. Statistical analyses were conducted by one-way ANOVA, ***p ≤ 0.001, ****p ≤ 0.0001.

The level of exposure to heavy metals and their subsequent uptake is not linear, especially in organisms known to bioaccumulate metals. Whilst the process of bioaccumulation is well-studied in macro-invertebrates (Huang et al., 2021) the microscopic nature of *C. elegans* has, until recently, prohibited the measurement of metals in individual nematodes. Here we were able to quantify the accumulation of zinc and cadmium in individual wild-type and metallothionein-knockout worms by X-ray fluorescence imaging (XFI) and laser ablation-inductively coupled plasma-mass spectrometry (LA-ICP-MS) (Fig. 1 and Fig. S1). XFI allowed the accumulation of zinc to be quantified at high resolution and with elemental specificity, this was not feasible for cadmium due to partly overlapping fluorescence bands arising from calcium. In contrast, LA-ICP-MS returned negligible background signal for the cadmium isotope ^111^Cd and thus was applied to measure the accumulation of cadmium in individual worms. The zinc (150 μM) and cadmium (30 μM) doses chosen for this study are based on a previous publication (Anderson et al., 2001) and reflect equi-toxicity, at least in terms of reproductive performance, a key lifecycle endpoint and sensitive indicator of metal toxicity (Pei et al., 2022) and are within the concentrations found in natural environments. It should be noted that, prior to any quantification, the intestinal lumen was allowed to clear to ensure the removal of free-floating metals. Hence the observed increase corresponds to metals taken up by the gut cells after ingestion via the mouth. Basal zinc levels (in the absence of added zinc) were comparable in wild-type (8.2 ± 1.5 μg/cm^3^) and the metallothionein-knockout mutants *mtl-1* (tm1770) (7.5 ± 1.7 μg/cm^3^) and *mtl-2* (gk125) (6.3 ± 1.1 μg/cm^3^). Worms raised from L1 to L4 stage (for 48 h) on agar and food supplemented with 150 μM zinc were characterised by a quantifiable increase in metal body burden within the posterior alimentary canal in wild-type (24.2 ± 3.1 μg/cm^3^) which was even more pronounced in *mtl-1* (tm1770) (62.3 ± 12.3 μg/cm^3^) and *mtl-2* (gk125) (47.5 ± 10.7 μg/cm^3^). Accordingly, the deletion of MT, in particular the knockout of *mtl-1*, resulted in a statistically significant increase in zinc accumulation (Fig. 1A). Our quantification from single worms aligns well with the measurements derived previously from bulk preparations where 5000 nematodes were exposed to 340 μM Zn (Zeitoun-Ghandour et al., 2010). Closer inspection of the localisation of zinc in single worms suggests that the accumulation is not uniform throughout the gut, but occurs in the form of “hotspots”. Due to limited resolution, we were not able to substantiate whether the zinc hotspots are limited to granules in the intestinal cells, which was previously shown (Roh et al., 2012), or in fact are co-localised with/in the nuclei (compare the zinc-exposed *mtl-1* mutant, Fig. 1A, to the visualisation of P*mtl-1*:GFP and P*mtl-2*:mCherry expression (see next section), which was restricted to the nucleus due to the inclusion of a Nuclear Localisation Sequence (NLS) (Fig. 2B)). This notion warrants further investigation, possibly via the inclusion of a nuclear stain (e.g., DAPI or Hoechst) or through use of a nuclear GFP expressing mutant strain.

When MTL-1 was overexpressed via an extrachromosomal array (*Pmtl-1*:MTL-1), the signal derived from metallothionein-bound Zn was amplified, revealing an accumulation around the anal region of the worm. Together with the increased body burden in the knockout mutant, this suggests that in *C. elegans* MTL-1 might be involved in the transport and excretion of excess zinc (Fig. S1).

The quantification of cadmium was expressed as total ion counts (cts) per pixel across the body of the worm, which increased upon exposure to 30 μM Cd in wild-type from a baseline level of 15.8 ± 2.6 cts to 276.1 ± 20.2 cts in exposed animals. The ability of wild-type and *mtl-1* (tm1770) to accumulate Cd was statistically indistinguishable (*mtl-1* (tm1770): 247.8 ± 39.6 cts), however Cd levels were significantly reduced in worms lacking *mtl-2* (*mtl-2* (gk125): 140.0 ± 19.7 cts) (Fig. 1b). Subtracting the cts from unexposed worms from the values obtained from exposed animals revealed that exposed *mtl-1* (tm1770) accumulated 93.3% but *mtl-2* (gk125) only 50.1% of cadmium compared to their wild-type counterpart. This highlights that the absence of MTs, in particular MTL-2, suppresses the bioaccumulation of cadmium. The relative (marginal) reduction in the ability of the *mtl-1* mutant to accumulate Cd was also reported by (Zeitoun-Ghandour et al., 2010) but contradicts their findings linked to the *mtl-2* knockout which, in their bulk sample analysis, suggested that *mtl-2* mutants bioaccumulated more Cd than wild-type (Zeitoun-Ghandour et al., 2010). We are not able to hypothesise whether this is due differences in experimental procedures (dosing and exposure/washing routines) or quantification technologies (measurement in intact individual worms vs nitric acid digested bulk preparations). However, the results obtained from single worm analyses suggest that MTs in *C. elegans* are involved in either an active elimination (excretion) of Zn or the transport of Cd into storage granules (e.g., coelomocytes).

### *3.2* *Basal and metal-induced transcription of* mtl-1 *and* mtl-2 *differ*

To correlate metal accumulation with gene expression, the relative and absolute transcriptional response of both MTs was quantified in nematodes exposed to zinc or cadmium. We created single copy integrated transgenic worm strains that express either Green Fluorescent Protein (GFP) driven by the promoter of *mtl-1* (P*mtl-1*:GFP) or Red Fluorescent Protein (RFP) driven by the *mtl-2* promoter (P*mtl-2*:mCherry) (Fig. 2A and Fig. S2). The transgenic constructs were designed to contain a nuclear localisation signal (NLS) which concentrated the fluorescent signal within a small defined space and facilitated easy *in vivo* visualisation. In the absence of added metals, P*mtl-1*:GFP was constitutively expressed in the pharyngeal cells, which supports previous reports (Swain et al., 2004; Baesler et al., 2021). Note, due to the construction of a single-copy transgene, fluorescent output was limited when visualised at low magnification (10x). Closer inspection of the pharynx at 100× magnification (Nikon TE2000 and Confocal A1R) however identified that P*mtl-1*:GFP expression was not just restricted to the posterior cells of the second pharyngeal bulb, but intriguingly was also present in all muscle cells of the anterior bulb, a new finding owing to advances in high-resolution confocal (Fig. 2A). No notable change in (enhanced) *mtl-1* expression was observed in the pharynx of Cd or Zn exposed nematodes (data not shown). No P*mtl-2*:mCherry was observed in the pharynx of unexposed or exposed animals (Fig. 2A). Conversely, a distinct basal expression of P*mtl-2*:mCherry, but not P*mtl-1*:GFP, was apparent in the gut cells of unexposed worms. Upon challenging the worms with cadmium or zinc, both P*mtl-1*:GFP and P*mtl-2*:mCherry were induced in the gut. In both cases, cadmium was a more potent inducer of MT expression than zinc. The change in P*mtl-1*:GFP fluorescence was generally more dynamic than that of P*mtl-2*:mCherry (Fig. 2B and C). Isoform-specific quantification of MT transcription by quantitative PCR (qPCR) confirmed the induction of MTs, which was highly significant in worms exposed to cadmium (Fig. 2D). We confirmed that an exposure from L1 to L4 stage (48 h) resulted in a robust induction of *mtl-1* and *mtl-2.* The relative magnitude of induction of *mtl-1* was higher than that of *mtl-2* (Cd exposed worms: *mtl-1* and *mtl-2* induction 40.5 ± 1.5 and 15.8 ± 0.5 fold, respectively; Zn exposed worms: *mtl-1* and *mtl-2* induction 5.4 ± 0.4 and 4.0 ± 0.5 fold, respectively) (Fig. 2D).

The fold-change of *mtl-1* and *mtl-2* in metal exposed worms, as determined by qPCR, is however a relative value which does not consider differences in baseline expression. Given that the expression of P*mtl-1*:GFP was very low in the absence of metal challenge, we determined the absolute copy number of *mtl-1* and *mtl-*2 by qPCR which confirmed that the baseline expression of *mtl-1* was two orders of magnitude lower than that of *mtl-2*. Although the induction potential of *mtl-1* significantly exceeded that of *mtl-2*, the copy number of *mtl-1* (even in cadmium exposed worms) remained well below the copy number of *mtl-2* in unexposed worms (Fig. 2E).

To explore the dynamics of expression upon a shorter (4 h) exposure to a higher dose, L1 worms (P*mtl-1*:GFP and P*mtl-2*:mCherry) were allowed to develop to L4 stage on control plates, then transferred for 4 h to plates supplemented with 500 μM Cd or Zn, a lethal dose if administered chronically (Fig. S3). The transcriptional response to the acute toxic metal challenge resulted in pronounced induction of *mtl-1* (Cd: 14.9 ± 1.1 fold, Zn 19.0 ± 1.2 fold) but only a modest change in *mtl-2* expression (Cd: 1.7 ± 0.1 fold, Zn 1.7 ± 0.2 fold).

Thus, the fold-change induction of *mtl-1* was markedly higher compared to *mtl-2* in both acute and chronic exposure settings, highlighting that *mtl-1* expression is transcriptionally more responsive to metal exposure than *mtl-2*. Furthermore, it is noteworthy that the expression of *mtl-1* was higher in worms challenged with a short acute exposure to high levels of Zn than that in worms exposed to the same Cd concentration (Fig. S3). However, in both chronic and acute exposures, the total number of *mtl-1* transcripts remained below the copy number of *mtl-2*, as the former has a very low baseline expression whilst the latter is characterised by a very high basal expression.

### *3.3* *The expression of* mtl-1 *is modulated by* mtl-2 *status (but not vice versa)*

**Fig. 3:**
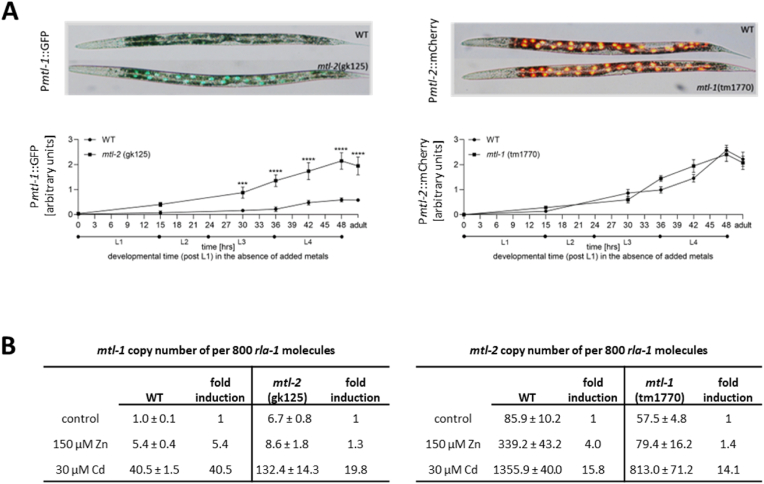
Metallothionein promoter activity in a wild-type and metallothionein mutant background. (A) The transgenic worms introduced in Fig. 2 were crossed with metallothionein mutants and the fluorescence quantified from L1 stage to adult stage, n = 10 per strain. (B) The copy number of *mtl-1* and *mtl-2* was normalised to 800 *rla-1*, to set the basal expression of unexposed wild-type to 1. Statistical analyses were conducted by one-way ANOVA, *p ≤ 0.05, **p ≤ 0.01, ****p ≤ 0.0001.

We crossed P*mtl-1*:GFP with the *mtl-2* (gk125) knockout and the P*mtl-2*:mCherry with the *mtl-1* (tm1770) knockout to explore the interplay between the two MTs (Fig. S2). Remarkably, even under control conditions (NGM plates without added metals), P*mtl-1*:GFP was visibly induced in the absence of *mtl-2*, as reflected by a strong increase in green fluorescence (Fig. 3A). In contrast, no visible change in P*mtl-2*:mCherry levels was noted in dependence of presence or absence of *mtl-1* (Fig. 3A).

This was corroborated by qPCR designed to quantify absolute copy numbers of *mtl-1* and *mtl-2* normalised to the invariant ribosomal housekeeping gene *rla-1.* As stated above, the copy number of *mtl-1* in the absence of a metal challenge was very low compared to that of *mtl-2* (Fig. 2E). The deletion of *mtl-2*, however, resulted in a 6.7-fold increase in *mtl-1* transcripts, notably in the absence of metal stress (i.e. on control plates). Furthermore, exposure to either Zn or Cd increased the *mtl-1* copy number in *mtl-2* (gk125) more pronouncedly than in the wild-type (Fig. 3B).

The opposite was observed in the case of *mtl-2* expression with/without *mtl-1* deletion. The high copy number of *mtl-2* at basal levels was reduced by 33% (from 85.9 to 57.5) in the *mtl-1* (tm1770) deletion strain. The induction of *mtl-2* upon exposure to Cd was similar in terms of fold change (wild-type 15.8, *mtl-1* (tm1770) 14.1 fold), but the copy number of *mtl-2* was considerably lower in *mtl-1* (tm1700) (813.0) compared to wild-type (1355.9). At this point in time, we are not able to offer an evidence-based mechanistic explanation of these observations, but this certainly warrants further investigations.

Interestingly, comparing the fold-induction upon metal exposure within the respective strains indicates that the deletion of either *mtl-1* or *mtl-2* results in a loss in Zn responsiveness of *mtl* expression (*mtl-1* expression upon Zn exposure: 5.4 fold in wild-type vs 1.3 fold in the *mtl-2* (gk125) and *mtl-2* expression upon Zn exposure: 4.0 fold in wild-type vs 1.4 fold in *mtl-1* (tm1770)). The deletion of *mtl-2* also reduced the Cd-mediated induction potential of *mtl-1*, but only by 50% (*mtl-1* expression upon Cd exposure: 40.5 fold in wild-type vs 19.8 fold in the *mtl-2* (gk125)). The deletion of one *mtl* gene therefore results in a transcriptional change of the other *mtl*. This may suggest that *mtl-1* and *mtl-2* work in concert to handle the homeostasis of toxic/essential metal via a mechanistic interdependency. Alternatively, this may just reflect an indirect effect, where the deletion of one *mtl* changes the metal status, balance and/or sensing ability within the worm, which in turn affects the expression of the other *mtl.*

The total *mtl* copy number (i.e., *mtl-1* and *mtl-2* combined) increased significantly in wild-type worms exposed to Zn and more so when exposed to Cd (Table S1A). However, despite the observed compensation by inducing *mtl-1* expression in a *mtl-2* knockout background, overall copies were reduced in both mutants, e.g. under basal conditions by 34% in *mtl-1* knockout worms and by 92% in *mtl-2* knockout worms, and under Cd exposure by 43% in *mtl-1* (tm1770) and by 91% in *mtl-2* (gk125) (Table S1B). The fact that metallothionein knockout worms are viable upon a metal challenge, highlights that metallothionein status is not an indispensable component of essential/toxic metal trafficking.

Previous reports have identified that *mtl-1* is constitutively expressed in the pharynx (Freedman et al., 1993; Swain et al., 2004), and our transgenic and qPCR results demonstrate for the first time that *mtl-2* is also constitutively expressed, however in the gut. The latter suggests that *mtl-2* is involved in essential metal handling in standard ambient conditions. However, if *mtl-2* is absent (e.g., in a mutant) or overwhelmed (through metal overload), *mtl-1* is upregulated in the gut. To what extent *mtl-1* can rescue the toxic outcome of excess metal will be explored in more detail later (see lifespan data).

Of course, transcription is only the initiator of the translation and it is the MTL proteins which engage in metal binding, transport, mobilisation of essential metals and detoxification of toxic metals. Studying structure and dynamics of the two proteins may help to explain the observations made so far and also provide insights into the origins of metal selectivity identified previously (Leszczyszyn et al., 2011). Exploring functional and structural differences between the 2 MT at the protein level was therefore the next step of the study.

### 3.4 The folding behaviours of MTL-1 and MTL-2 differ

**Fig. 4:**
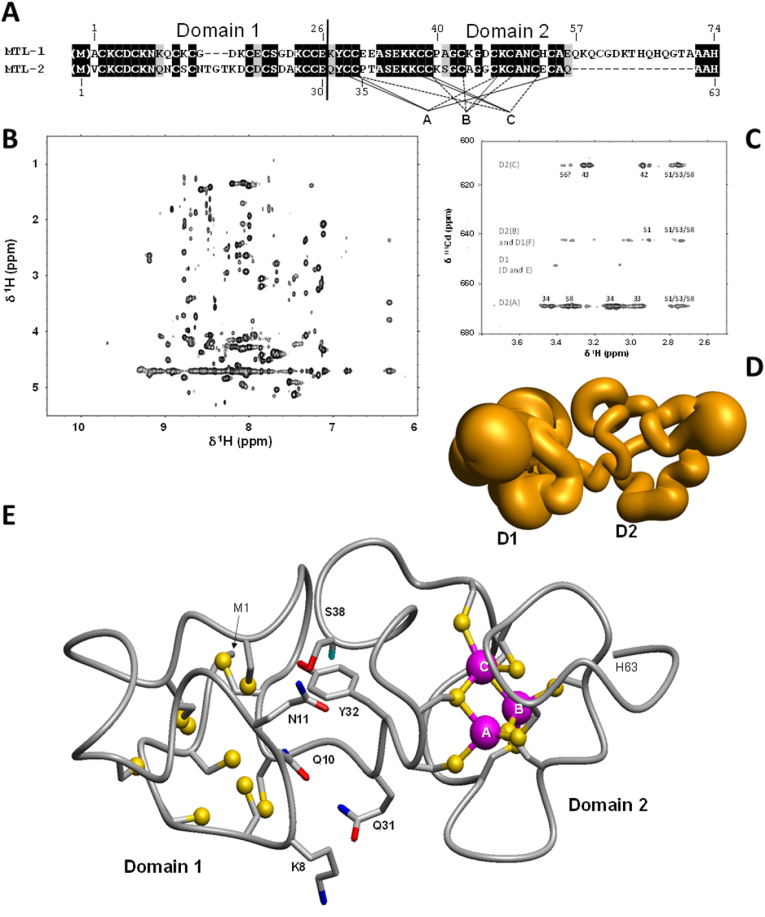
*C. elegans* MT sequences and solution structure of Cd_6_MTL-2. (A) Amino acid sequences of MTL-1 and MTL-2. Black highlights show conserved residues, grey highlights indicate semi-conservation. The numbering refers to the sequences of the mature overexpressed proteins, with the N-terminal Met cleaved in the case of Zn_7_MTL-1, but present in Cd_6_MTL-2 (31). (B) 2D [^1^H,^1^H] TOCSY spectrum of Cd_6_MTL-2 (1 mM protein, 50 mM Tris-D_11_, 100 mM NaCl, pH 7.0, 303 K). (C) 2D [^1^H,^111^Cd] HSQC spectrum of labelled ^111^Cd_6_MTL-2, using a coupling constant of ^3^*J* = 45 Hz (1 mM protein, 20 mM Tris-D_11_, 50 mM NaCl, pH 7.4, 298 K). Further HSQC spectra were acquired at 30 Hz or 60 Hz; the assignments shown are derived from all three spectra and are based on 2D [^1^H,^1^H] spectra of the same sample acquired under the same conditions. Cd-Cys connectivities derived from these spectra are shown in the lower part of (a). (D) Ensemble of 20 conformers for the ^1^H and ^111^Cd NMR spectroscopy-derived 3D structure of Cd_6_MTL-2. (E) Representative conformer showing a Cd_3_Cys_9_ cluster in domain 2 and residues engaged in inter-domain contacts.

The two MTs were expressed recombinantly in *E. coli* (in presence of either Zn^2+^ or Cd^2+^ in the culture medium), purified and analysed by mass spectrometry as previously described (Zeitoun-Ghandour et al., 2010). They share 63% sequence similarity across the first 59 residues, with all 18 cysteines that are present in MTL-2 fully conserved in MTL-1 (Fig. 4A). The 15-amino acid residues extension in MTL-1 features an additional Cys residue (Cys60) as well as two His residues (His65 and His67). In addition, Glu57 in MTL-2 is replaced by His53 in MTL-1; thus, MTL-1 harbours four additional residues with strong metal-binding potential. This very likely accounts for the higher metal:protein stoichiometry of MTL-1 (7 divalent metals compared to 6 in MTL-2) [30, 31, 49). We and others have also shown that MTL-2 has a clear preference for Cd^2+^, but for MTL-1 the situation is more complicated. As is the case for all MTs, the thermodynamic stability of Cd_7_MTL-1 exceeds that of Zn_7_MTL-1 ((Hall et al.) also see Fig. 6D); therefore it would be misleading to deem MTL-1 to be “zinc-specific”. However, we have also demonstrated that when both proteins are simultaneously present *in vitro*, Cd^2+^ partitions overwhelmingly into MTL-2, leaving MTL-1 largely populated with Zn^2+^ (Pei et al., 2022), in full accordance with measured affinity constants: As previously stated, the average stability constant for Cd_7_MTL-1 (log *K* = 13.1) is almost two orders of magnitude lower than that of Cd_6_MTL-2 (log K = 15.0) (Hall et al.). These considerations emphasise the important concept of relative affinities (Foster and Robinson, 2011).

To explore the structural origins of these differences in metal selectivity, we prepared all four combinations of protein and metal ion for analysis by NMR spectroscopy. As previously observed (Hall et al.), variations in cleavage of the N-terminal methionine were dependent on the metal ion supplied in the culture medium; the residue numbering shown (Fig. 4A) hence refers to the actual species that were used for the subsequent structural studies by solution NMR.

Intriguingly, the metal preferences outlined above were to some degree also reflected in the folding behaviour of the proteins: Zn_6_MTL-2 gave a poorly dispersed 1D ^1^H NMR spectrum (Fig. S4), and acquisition of 2-dimensional data was abandoned. In contrast, Cd_6_MTL-2 (Fig. 4B and Fig. S4) yielded high-quality 1D and 2D ^1^H spectra suitable for sequential assignment (Table S2). Together with determination of Cd-Cys connectivities by heteronuclear [^1^H,^111^Cd] NMR spectroscopy (Fig. 4C), this allowed the determination of a (partial) 3D structure (Fig. 4D and E and Fig. S5). The protein folds into two domains, each with nine Cys residues. A two-domain structure had been proposed previously based on biophysical studies of truncated constructs (Zeitoun-Ghandour et al., 2011). Unusually for two-domain MTs, it was possible to define the interface and mutual orientation of the two domains. Although not unprecedented (Baumann et al., 2017), this is a rare occurrence. In the present case, this is owed to the linker between the domains being short and several inter-domain NOE crosspeaks being present. Of particular importance for the domain interface is Tyr32 (conserved or semi-conserved (Phe) in all related MTs from a range of *Caenorhabditis* species; Fig. S6) that is buried at the domain interface (Fig. 4E). Typically, aromatic residues (other than histidine) are rare in MTs; it is thought that the metal-thiolate clusters in MTs perform an analogous task in stabilising ordered protein structure. The multiple NOE interactions for the aromatic protons of Tyr32 (Fig. S7) highlight its important role in ordering the domain interface. Of particular note is the large upfield shift (ppm) of the CHα proton of Ser 38 (2.204 ppm). This proton is located directly above the aromatic ring and is thus strongly shielded by its electron cloud. The remainder of domain 1 was much less well-defined due to incomplete sequential assignment, scarcity of NOE-derived distance restraints, and because the signals for ^111^Cd^2+^ bound to domain 1 were too broad to give sufficient information (Fig. 4C) – similar to the situation encountered in the β-domain of mammalian MT-3 (Faller et al., 1999). It is noteworthy that our 1D^111^Cd spectra of recombinant ^111^Cd_6_MTL-2 closely matched a published ^113^Cd NMR spectrum (You et al., 1999) in both chemical shifts and peak shape trends.

The structural data suggest that domain 2 harbours a classical M_3_Cys_9_ cluster, in which three metal ions and three bridging sulfur atoms form a six-membered ring, with the remaining six thiolates coordinating to a single metal ion. It is likely that the remaining nine sulfurs and three metal ions in domain 1 may form a similar cluster; the cause for the more dynamic nature of this domain is unclear – again similar to the situation in the β-domain of mammalian MT-3.

**Fig. 5:**
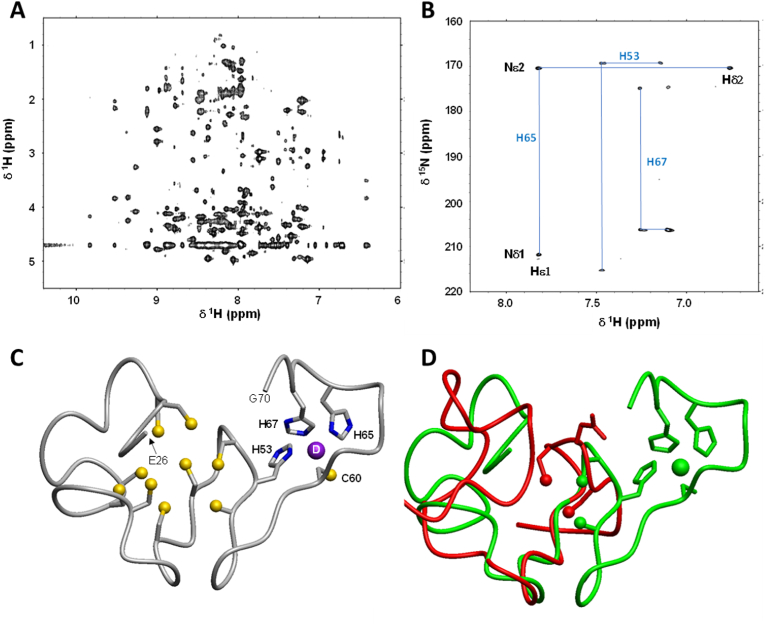
NMR solution structure of domain 2 of Zn_7_MTL-1 and comparison with MTL-2. (A) Fingerprint region of a 2D [^1^H,^1^H] TOCSY spectrum of unlabelled Zn_7_MTL-1 (1 mM protein, 20 mM NH_4_HCO_3_, 20 mM NaCl, pH 7.3, 303 K). (B) Extended region [^1^H,^15^N] HSQC spectrum of ^13^C,^15^N-labelled Zn_7_MTL-1 (0.5 mM protein, 25 mM NH_4_HCO_3_, 25 mM NaCl, pH 7.3, 303 K; ^2^*J* = 30 Hz), showing coupling between CHδ2 and CHε1 protons and Nδ1 and Nε2 nitrogens of histidine imidazole rings. Protons and nitrogens are labelled for His65. (C) Representative conformer (see Appendix Fig. S9 for the NMR ensemble; backbone atoms r.m.s.d. over residues 26 to 69 = 1.46 ± 0.30 Å). (D) Structural overlay between representative conformers of MTL-1 (green) and MTL-2 (red). This overlay is based on structural alignment of residues Lys44-Ala50 of MTL-1 with Ala48-Ala54 of MTL-2 (see Fig. 4a). The overlay illustrates that the overall protein folds of domain 2 in these two proteins differ dramatically, despite complete conservation of the first nine metal-binding cysteines. The sulfurs of Cys 56 and 58 (MTL-2) and Cys 52 and 54 (MTL-1) that flank either Glu57 or His53 are shown as spheres; their alternative positioning imposed by His53 being part of the mono-nuclear site G is evident. (For interpretation of the references to colour in this figure legend, the reader is referred to the Web version of this article.)

The folding behaviour of MTL-1 was also metal-dependent, but in perfect opposition to that of MTL-2. Here, the Zn_7_MTL-1 complex gave high-quality NMR spectra (Fig. 5A and Fig. S4). Although at a first glance, the 1D spectra of either Cd_7_MTL-1 or Cd_6_ZnMTL-1 had a reasonable dispersion (Fig. S4), backbone NH peaks were quite broad, and the corresponding 2D spectra were afflicted by a lack of cross-peaks for residues from both domains, with sequential assignment possible for several domain 1 residues only. Similarly, multiple and varied attempts to generate ^111^Cd-labelled samples (either purely cadmium-loaded or in mixtures with zinc) that would yield resolved ^111^Cd NMR spectra failed.

**Fig. 6:**
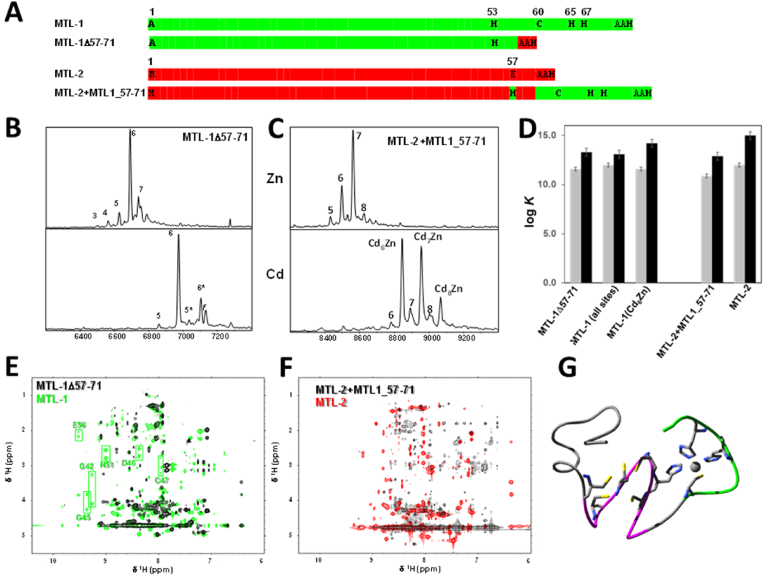
Cross-talk between the C-terminal extension and the remainder of domain 2. (A) The MTL-1 “tail-deletion” lacks residues Gln57-Ala71, and the MTL-2 “tail-insertion” mutant comprises 15 addition residues, plus the C-terminal AAH motif that is common to both MTs. In addition, Glu57 of MTL-2 was mutated to histidine, ensuring that all four metal-binding residues of the mono-nuclear site G are present in the mutant. (B) Native ESI-MS spectra (25 μM protein in 10 mM ammonium acetate, pH 7.4; deconvoluted onto a true mass scale) of the MTL-1 tail deletion mutant expressed in the presence of Zn^2+^ (upper panel) or Cd^2+^ (lower panel). The numbers indicate the number of metal ions bound. The asterisk indicates forms that comprise the N-terminal methionine; all other peaks refer to forms where this methionine has been cleaved. *f* denotes formylation. (C) Native ESI-MS spectra (25 μM protein in 10 mM ammonium acetate, pH 7.4; deconvoluted onto a true mass scale) of the MTL-2 tail insertion mutant, expressed in presence of Zn^2+^ (upper panel) or Cd^2+^ (lower panel). See Appendix Table S8 for observed and theoretical masses. (D) Comparison of Zn^2+^ (grey bars) and Cd^2+^ (black bars) affinities between mutants (this work) and wild-type proteins (31, 42), determined by competition with 5F-BAPTA (10 mM Tris, pH 8.1). (E) Overlay of 2D TOCSY data for Zn_7_MTL-1 (green; 20 mM NH_4_HCO_3_; 20 mM NaCl, pH 7.3, 303 K) and Zn-MTL-1Δ57-71 (black; 20 mM Tris-D_11_, 50 mM NaCl, pH 7.3, 303 K, 700 MHz), indicating major structural changes in domain 2. (F) Overlay of 2D TOCSY NMR data for Cd_6_MTL-2 (red; 20 mM Tris-D_11_, 50 mM NaCl, pH 7.3, 303 K, 700 MHz) and the tail insertion mutant (black; 20 mM Tris-D_11_, 50 mM NaCl, pH 7.3, 303 K, 700 MHz), indicating widespread structural disorder in the latter. (G) Residues Lys44-Gln57 that are affected severely by tail (green) deletion in MTL-1 are highlighted in magenta (see Appendix Fig. S14 for a chemical shift perturbation plot). (For interpretation of the references to colour in this figure legend, the reader is referred to the Web version of this article.)

Therefore, structural studies focused on Zn_7_MTL-1. In this case, we also exploited labelling with ^15^N and ^13^C, which not only facilitated sequential assignment (Table S3), but also inspection of imidazole ^15^N signals and their coupling patterns with ^1^H (Fig. 5B). The patterns in the latter [^1^H,^15^N] HSQC spectrum inform on tautomerisation state, with – additionally – ^15^N chemical shifts over 200 ppm being indicative of metal-coordinated imidazole (Urbani et al., 1998). These data suggest that His53 and His65 bind zinc via their δ1 nitrogen, whereas His67 binds via the ε2 nitrogen. The pH-dependence of the chemical shifts of the Hε1 and Hδ2 protons of these three histidine residues is also indicative of metal binding (Fig. S8): They remain virtually unchanged between pH 8 and 5.5, whilst at lower pH, the protein becomes unfolded, with His resonances no longer distinguishable from each other. The fourth, C-terminal, His residue (His74) was not observed in the extended HSQC region, presumably due to unfavourable dynamics, but in 1D ^1^H spectra, its Hε1 and Hδ2 protons showed pH behaviour typical for a non-coordinated histidine with a typical p*K*_a_ value around 6–7 (Fig. S8). Bofill et al. (Zeitoun-Ghandour et al., 2011) have provided independent corroboration that three (out of four) His residues in Zn_7_MTL-1 are indeed involved in zinc binding and have also shown that the C-terminal His residue in MTL-2 (His 63) has no major role in metal binding.

Like for Cd_6_MTL-2, domain 1 of Zn_7_MTL-1 appeared to be more dynamic than domain 2, with several residues remaining unassigned. Distance and angle (from 3-bond coupling constants derived from an HNHA experiment) restraints from the remaining residues were also too few to determine the backbone fold of domain 1 with sufficient definition. We therefore restricted the structure determination to domain 2 only. This gave a (for an MT) well-defined ensemble, even without the inclusion of any metal-ligand restraints (Fig. S9). This was also the case for the majority of the 15-residue C-terminal insertion; indeed, the three additional histidine residues and Cys60 showed a number of inter-residue NOE crosspeaks that yielded side-chain conformations close to those required to form a mono-nuclear His_3_Cys zinc site (Fig. S10). Whilst metal-coordinating histidines have been encountered in several MTs, these have so far involved either Cys_3_His sites in bacterial MTs (Leszczyszyn and Blindauer, 2010) or a Cys_2_His_2_ site in certain plant MTs (Leszczyszyn et al., 2010; Peroza and Freisinger, 2007). Thus, the His_3_Cys site found in Zn_7_MTL-1 is the first such site observed in a metallothionein. His_3_Cys sites, albeit formed by very different sequence motifs, are present in a small number of other structurally characterised proteins, including bacterial and archaeal tRNA-synthases and -editing proteins, a viral envelope glycoprotein complex, and a human histone lysine N-methyltransferase (Table S4). It seems likely that these sites stabilise protein structure. Most notably however, a His_3_Cys site that senses Zn^2+^ is present in an *E. coli* diguanylate cyclase (Zähringer et al., 2013).

For the remainder of the domain, it was not possible to predict metal-ligand connectivities. A comparison of the backbone folds for domains 2 of MTL-1 and MTL-2 (Fig. 5D) indicates large differences; therefore inference of the metal-thiolate cluster structure from MTL-2 for MTL-1 would not be appropriate. Closer inspection of backbone conformations (Fig. S11) indicates that Φ and Ψ angle combinations are only similar between the two proteins for the stretch between Cys39/Cys43 and Cys 49/Cys 53, with more or less large differences observed in the adjacent N- and C-terminal portions. In the N-terminal portion, this includes major differences seen for the fully conserved ^33/37^ASEKK^37/41^ motif. This may initially be surprising, but structural differences in this stretch are also reflected in significant differences in chemical shifts for some of the residues (Fig. S12). We observe that this conserved stretch is preceded by a short portion where the amino acids are not conserved: EE in MTL-1 and PT in MTL-2. The EE motif is fully conserved in MTL-1 homologues, the PT motif is an ST motif in other MTL-2 homologues (Fig. S6).

In addition, a further sequence deviation occurs after Cys39/43, with PA in MTL-1 and KS in MTL-2. The PA motif is fully conserved in MTL-1 homologues, but the KS motif is not (Fig. S6). The non-conservation of proline residues between MTL-1 and MTL -2 in this region seems noteworthy, as proline residues impose particular backbone conformations. However, apart from large differences between the Ψ angle for the E31/P35 pair and the Φ angle for the K41/S45 pair (Fig. S11), there is no clear indication that these proline residues are the major determinants of structural differences. At the C-terminal end, the mono-nuclear site G impacts on the relative location and orientation of the two cysteine residues that bracket His53 in MTL-1 or the equivalent Glu57 in MTL-2 (see Fig. 5D). This may account at least in part for the structural differences in the C-terminal portion of domain 2.

We suggest that these unexpectedly large differences in backbone conformation throughout domain 2 may be at the root of the observed metal-specific folding. We next explored the role of the C-terminal extension in MTL-1 in more detail, in particular its role in metal selectivity.

### 3.5 The C-terminal extension in MTL-1 communicates with the entire domain 2

Owing to its soft character, Cd^2+^ shows a pronounced preference for thiolate sulfurs over oxygen or nitrogen ligands, in contrast to borderline Zn^2+^, which is much more promiscuous. We have previously determined the affinities of MTL-1 and MTL-2 for Zn^2+^ and Cd^2+^, including those for the mixed-metal form Cd_6_ZnMTL-1, which is the major form isolated when MTL-1 is expressed in presence of Cd^2+^ in the culture medium (Zeitoun-Ghandour et al., 2010; Leszczyszyn et al., 2011). This allowed the determination of a Zn^2+^-binding constant for the mono-nuclear His_3_Cys site G in MTL-1 as log *K* = 11.6 ± 0.1, which is only marginally smaller than the average for the ZnCys_4_ sites (12.0 ± 0.1). The comparisons between the Cd^2+^-affinities of Cd_7_MTL-1 and Cd_6_ZnMTL-1 gave an estimate for the Cd^2+^-binding constant of the 7th binding site in Cd_7_MTL-1 as log *K* = 7.6 ± 1.1. This is over three orders of magnitude lower than that for Zn^2+^ – and over six orders of magnitude lower than those for the CdCys_4_ sites in these proteins (average log *K* ranging from 14.2 to 15.0). Judging from the misfolding observed for Cd_7_MTL-1, it is doubtful that Cd^2+^ even binds to site G in a similar manner to Zn^2+^, but nonetheless the low estimated log *K* can be considered an upper limit for the affinity of the His_3_Cys for Cd^2+^. Such a low affinity for a His_3_Cys site would be in keeping with expectations based on the hard-and-soft-acids-and-bases principle, as observed for zinc-finger peptides (Krizek et al., 1993). Hence, irrespective of whether it features in Cd_7_MTL-1, the mono-nuclear site G is decisively zinc-specific, a conclusion that is also supported by the dominant formation of the Cd_6_ZnMTL-1 form when the protein is metallated *in vivo* (in *E. coli*). Nonetheless, the average affinity for the remaining six Cd^2+^ ions in Cd_6_ZnMTL-1 is still almost an order of magnitude lower than that for Cd_6_MTL-2 (Leszczyszyn et al., 2011). In order to explore how the mono-nuclear His_3_Cys site may affect metal affinity and folding of the remainder of domain 2, we generated an MTL-1 “tail-deletion” mutant (MTL-1Δ57-71; Fig. 6A) and an MTL-2 “tail-insertion” mutant (MTL-2+MTL-1_57–71; Fig. 6A), and expressed these proteins in the presence of either Zn^2+^ or Cd^2+^. The products were analysed by native ESI-MS (Fig. 6B and C) and 1D and 2D ^1^H NMR (Fig. 6E and F and Fig. S13), and their Zn^2+^ and Cd^2+^ affinities were determined by competition with 5F-BAPTA and compared to the corresponding wild-type data (Fig. 6D (Hall et al.; Pei et al., 2022);).

The MTL-1 tail deletion mutant yielded predominantly species with 6 Zn^2+^ or Cd^2+^ bound, with minor ESI-MS peaks for both under- and over-metallated species for Zn-MTL-1Δ57-71, and only a very small proportion of Cd_5_MTL-1Δ57-71. In contrast, the metallation state of the MTL-2 tail insertion mutant was considerably more inhomogeneous, with between 5 and 8 Zn^2+^ ions bound when expressed in the presence of Zn^2+^. The species observed when this mutant was expressed in the presence of Cd^2+^ contained between 7 and 9 metal ions, with the two major species containing one Zn^2+^ ion and 6 or 7 Cd^2+^ ions. These Zn-containing major species suggest that the additional 4 ligands are still capable of forming a mono-nuclear His_3_Cys site that preferentially binds Zn^2+^.

The affinities (expressed as log *K*) of both mutants towards either Zn^2+^ or Cd^2+^ were, to various degrees, smaller than those of the respective wild-type, with tail deletion having a smaller effect (−0.4 for Zn^2+^ and −0.9 for Cd^2+^) than tail insertion (−1.1 for Zn^2+^ and −2.1 for Cd^2+^). This suggests that the mononuclear site is not independent of the remainder of the protein.

Both ESI-MS and affinity data indicate that deletion of the tail in MTL-1 is better tolerated than insertion of the tail into MTL-2. Assessment of folding behaviour by ^1^H NMR spectroscopy essentially reflects this conclusion: neither the Zn- nor the Cd-form of the tail insertion mutant were well folded, with most NH resonances being broad and concentrated between 8.0 and 8.5 ppm (Fig. 6F and Fig. S13).

In contrast, both Zn- and Cd-MTL-1Δ57-71 were (at least) partially folded. For Zn-MTL-1Δ57-71, residues Lys 3 to Cys43, Asp 46, and Ala 57 to His 59 could be assigned with the help of the wild-type assignments (with Ser20-Cys 24 remaining unassigned as for the wild-type). The inability to identify resonances for almost all residues from Lys44 to Glu 56 (highlighted in magenta in Fig. 6G) indicates that this stretch is severely perturbed by the absence of the tail in Zn-MTL-1Δ57-71. Furthermore, a comparison of backbone NH and CHα shifts for the assigned residues in wild-type Zn_7_MTL-1 and Zn-MTL-1Δ57-71 (Fig. S14) shows that in fact most residues following Tyr28 – *i.e.* the entirety of domain 2 – are affected, to some degree, by the absence of the tail.

Intriguingly however, for Cd-MTL-1Δ57-71, assignment was additionally possible for residues Lys44 to His53, albeit with considerable deviations in chemical shifts between this form and the Zn-bound wild-type (Fig. S15) – suggesting structural differences. The excellent resolution of the respective crosspeaks (Fig. S15) and the resulting facility with which these residues could be assigned suggests that this part of the structure is well-ordered in Cd-MTL-1Δ57-71 – in contrast to Cd_7_MTL-1, Cd_6_ZnMTL-1 and Zn-MTL-1Δ57-71. From this observation, it is tempting to speculate that the presence of the Zn-His_3_Cys site negatively impacts the ability of domain 2 to fold well with Cd^2+^ – which may also account for the reduced affinity for Cd^2+^ in Cd_6_ZnMTL-1.

Summarily, the analysis of these four protein preparations suggests that firstly, the mono-nuclear site in MTL-1 does not operate in isolation, but interacts with the remainder of the domain. It is conceivable that at least some of this “structural communication” is transmitted through the metal-thiolate cluster. Secondly, analysis of NMR data including the partial sequential assignment of the MTL-1Δ57-71 mutant implies that the tail promotes ordered structure in the presence of Zn^2+^, but disorder in the presence of Cd^2+^. We suggest that this opposing effect may play a major role in explaining the metal selectivity of MTL-1, and its preferential metallation with Zn^2+^ when present together with MTL-2 (Leszczyszyn et al., 2011).

### *3.6* *Deletion of* mtl-2 *significantly increases the cadmium-toxicity mediated reduction in lifespan*

Chemical toxicity rankings derived from worms align well with studies conducted in mammalian counterparts (Hunt, 2017), providing confidence that the power of predictive mechanistic toxicology can be extrapolated across evolutionary boundaries. *C. elegans* lifespan is a powerful endpoint that evaluates the relative toxicity of chemical exposure within a rapid timeframe. Having said that, due to the rapid life-cycle/generation time, spontaneous mutations can occur more readily and can introduce variables that can affect aging rates (Gems and Riddle, 2000). Although the strains used in this present study were back-crossed four times, the metallothionein deletion strains were generated in and sourced from different laboratories and this alone may (or may not) explain divergence in median lifespan of unexposed wild-type and the metallothionein deletion strains (Table S5). However, the comparison of the relative response to metal exposure within each strain is possible and can help define the importance of metallothionein status.

**Fig. 7:**
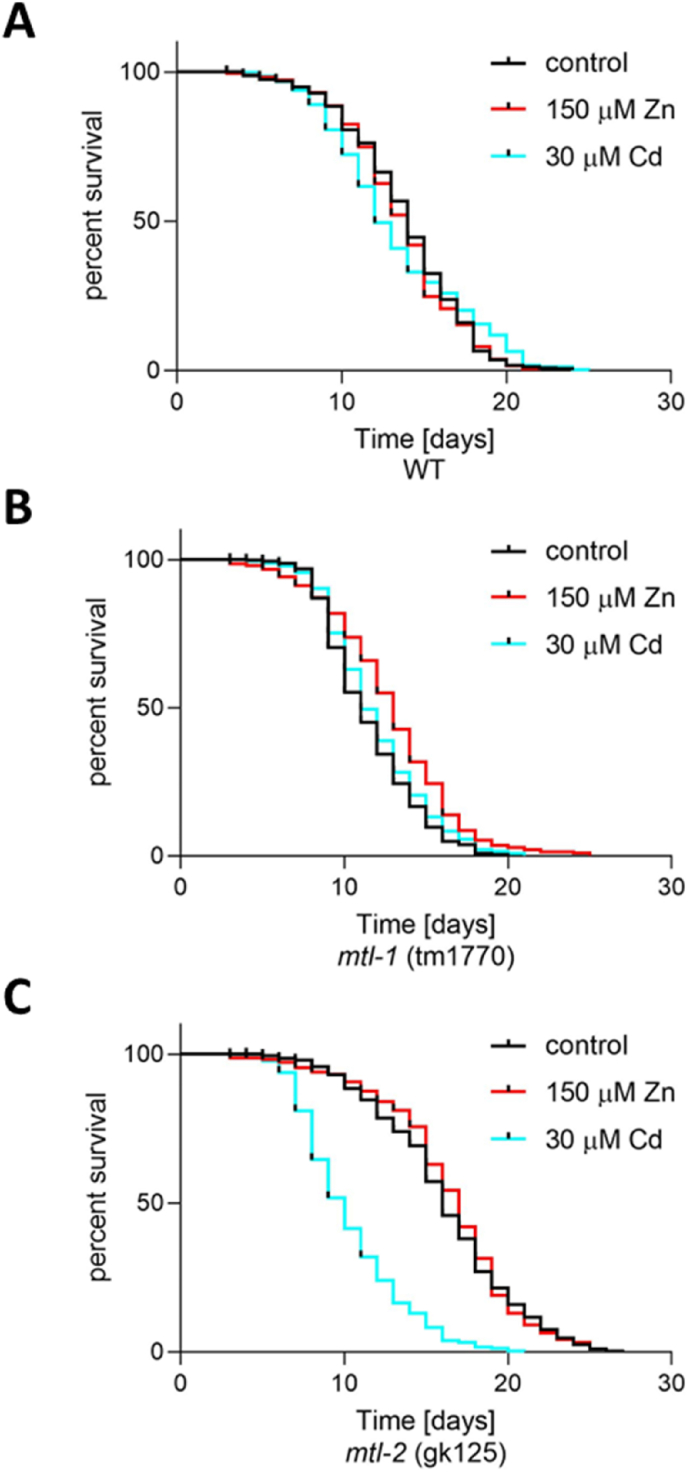
The metal exposure effects on lifespan. Lifespan was studied in (A) wild-type worms, (B) *mtl-1* (tm1770) knockout worms and (C) *mtl-2* (gk125) knockout worms raised either on NGM plates or NGM plates supplemented with 150 μM Zn or 30 μM Cd. For each condition, 400 synchronised nematodes were monitored from L1 until death. Every 24 h surviving nematodes were determined. The data were plotted as Kaplan-Meier survival graphs and statistical analysis was performed using the Logrank (Mantel-Cox) Test for comparison of survival curves (Appendix Table S5).

A chronic supplementation of 150 μM zinc did not impact the median survival and overall lifespan of wild-type worms. The exposure to 30 μM cadmium reduced median survival by two days, but overall, the survival curve was similar to their unexposed counterparts. The lifespans of unexposed and exposed animals were statistically indistinguishable. This suggests that wild-type worms are able to cope with excess zinc and cadmium, at least as far as lifespan is concerned and the doses tested (Fig. 7A, Table S5). The zinc and cadmium threshold concentrations needed to significantly impact lifespan in wild-type worms are, at this point, not known. Likewise, the deletion of *mtl-1* did not impair the median lifespan of cadmium-exposed animals, but the addition of zinc improved lifespan in a statistically significant manner (median lifespan *mtl-1* (1770): unexposed and cadmium-exposed 11 days; zinc-exposed 13 days) (Fig. 7B, Table S5). A highly pronounced reduction in lifespan was however observed when the *mtl-2* mutant was challenged with 30 μM Cd, characterised by a highly significant increase in death from day 8 onwards (median lifespan *mtl-2* (gk125): unexposed 16 days; Cd-exposed 10 days).

## 4 Conclusion

Overall, this study uncovered for the first time the interplay between the two *C. elegans* metallothionein isoforms. The metal accumulation patterns were metallothionein-dependant, where the deletion of *mtl-1* resulted in a more pronounced increase in Zn and the deletion of *mtl-2* in a sharp reduction in Cd accumulation (compared to wild-type). We showed that *mtl-1* has a significantly more dynamic gene expression response towards divalent metal exposure, compared to *mtl-2*; however, this is accompanied by very different base-line levels. Whilst *mtl-2* is constitutively expressed in the gut and induced further upon metal exposure, *mtl-1* is constitutively expressed in the pharynx but only transcriptionally activated in the gut upon metal challenge. The deletion of *mtl-2* results in a strong transcriptional induction of *mtl-1* in the gut, however *mtl-2* expression is less impacted by the deletion of *mtl-1*. Whilst the worm seems to be able to cope with a metal challenge in the absence of *mtl-1*, the significantly shortened lifespan of a cadmium-exposed *mtl-2* mutant underlines the presence of a significant toxic outcome.

These findings regarding physiological functions correlate well with the differences in structural, dynamic and metal-binding properties of the proteins. Both proteins can bind both metal ions, but protein folding is metal-dependant, especially in the C-terminal domains: MTL-1 folds well when bound to seven Zn^2+^ ions, but shows structural disorder in presence of Cd^2+^, whilst MTL-2 is well-folded when bound to six Cd^2+^ ions, but more dynamic when bound to Zn^2+^. The disfavoured binding of Cd^2+^ in the C-terminal domain of MTL-1 is likely to be at least partially mediated by the zinc-specific mono-nuclear His_3_Cys site G that is formed by the 15-amino acid residue insert that is specific to MTL-1. There are no structural precedents for this new site, although other sequence motifs are known to form zinc-bound His_3_Cys sites (Table S4). At least one of these sites is known to be “zinc-sensory”, which raises the question whether this could also be the case for MTL-1, especially in cells with constitutive expression. Secondly, given that zinc binding to this site hampers cadmium binding to the remainder of the domain, this phenomenon could be described as a case of allosteric inhibition of cadmium binding by zinc.

Taken together, it may be envisaged that the less complex but highly expressed MTL-2 is sufficient to handle zinc ions in the gut in a normal physiological environment. If the system is overwhelmed by excessive metal ions (Zn or Cd), which could be due to exposure (exogenous metal overload) or due to the loss of function of an abundant metal chelator such as MTL-2, the expression of MTL-1 is activated, and can – as far as zinc homoeostasis is concerned – compensate for the absence/unavailability of MTL-2. MTL-2 is however required to guard the worm from the toxic consequences of a chronic exposure to cadmium. As previous work has shown, when zinc and cadmium are simultaneously present, cadmium will partition overwhelmingly into MTL-2, which may leave MTL-1 to take over zinc handling.

Metal metabolism is of course a highly dynamic process involving many players including, but not limited to, metallothioneins. This is, perhaps, the limitation of this study. Investigating gene/protein function in isolation provides information on specific mechanisms but ignores interactions and the control of metal homeostasis/detoxification is no doubt more intricate than a two-protein interplay.

In summary, *C. elegans* metallothioneins seem to be characterized, like other soil invertebrates (Stürzenbaum et al., 2001; Kowald et al., 2016; Dallinger et al., 1997; Beil et al., 2019) by unique isoform and metal-selective features that differ from the “classical” MTF-1 mediated pathway in higher organisms. Here, we uncover the presence of two inducible metallothionein isoforms with different metal selectivity and inducibility which play a prominent role in allowing *C. elegans* (and related species with two MTs; see e.g. Fig. S6) to maintain a specific response towards the exposure to essential zinc and toxic cadmium.

## Data and materials availability

All structural statistics are summarised in Appendix Tables 6 and 7, and Appendix Fig. S5 and Fig. S6. All final models were validated using the MolProbity (http://molprobity.biochem.duke.edu/) and WHATIF (https://swift.cmbi.umcn.nl/whatif/) servers. Chemical shifts are deposited in the BioMagResbank, accession numbers 51546 (Cd-MTL-2) and 34,749 (domain 1 of Zn-MTL-1). For both proteins, ensembles for the 20 best structures are deposited in the Protein Data Bank under pdb codes 8AP5 (Cd-MTL-2) and 8AQ9 (domain 1 of Zn-MTL-1).

## CRediT authorship contribution statement

**Yona J. Essig:** Data curation, Formal analysis, Investigation, Visualization, Writing – original draft. **Oksana I. Leszczyszyn:** Data curation, Formal analysis, Investigation, Visualization. **Norah Almutairi:** Data curation, Formal analysis, Investigation, Validation, Visualization. **Alexandra Harrison-Smith:** Data curation, Investigation, Validation. **Alix Blease:** Formal analysis, Investigation, Validation. **Sukaina Zeitoun-Ghandour:** Data curation, Investigation, Validation. **Sam M. Webb:** Methodology, Resources, Software, Visualization. **Claudia A. Blindauer:** Conceptualization, Data curation, Funding acquisition, Methodology, Project administration, Resources, Supervision, Visualization, Writing – review & editing. **Stephen R. Stürzenbaum:** Conceptualization, Data curation, Funding acquisition, Methodology, Project administration, Resources, Supervision, Visualization, Writing – review & editing.

## Declaration of competing interest

The authors declare that they have no known competing financial interests or personal relationships that could have appeared to influence the work reported in this paper.

## Data Availability

Data will be made available on request.
