# Supplementary material for: Juggling cadmium detoxification and zinc homeostasis: A division of labour between the two C. elegans metallothioneins
Source: Chemosphere. 2024 Feb;350:141021. doi: 10.1016/j.chemosphere.2023.141021 (PMC11134313; doi:10.1016/j.chemosphere.2023.141021)
Supplement: Multimedia component 1 [file mmc1.docx]

**Appendix Table S1.** Copy number of *mtl* transcripts normalized to unexposed wildtype *mtl-1* (a) and net *mtl* numbers in *mtl* mutants compared to wild type (b).

**
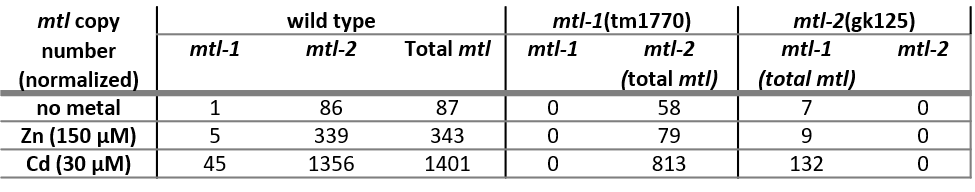

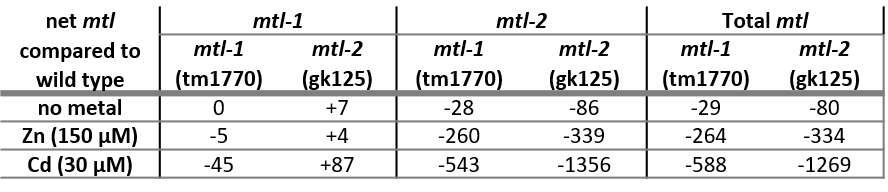
**

**A**

**B**

**Appendix Table S2.** ^1^H NMR chemical shifts for Cd_6_MTL-2.

| **Residue** | **H** | **H(α)** | **H(β)** | **H(γ)** | **H(δ)** | **H(ε)** |
| --- | --- | --- | --- | --- | --- | --- |
| **M1** | - | 4.583 | - | - | - | - |
| **V2** | 7.713 | 4.364 | 2.274 | 0.949 | - | - |
| **C3** | 8.426 | 4.185 | 3.394; 3.004 | - | - | - |
| **K4** | 9.689 | 4.204 | 2.059; 1.989 | 1.659 | - | 3.036 |
| **C5** | - | - | - | - | - | - |
| **D6** | - | - | - | - | - | - |
| **C7** | - | 4.196 | 2.581 | - | - | - |
| **K8** | 8.785 | 3.719 | 2.143 | 1.519 | 1.631 | 3.011 |
| **N9** | 7.445 | 4.916 | 3.010 | - | - | - |
| **Q10** | 9.061 | 4.096 | 2.018; 1.890 | 2.244 | - | - |
| **N11** | 8.039 | 4.773 | 2.929; 2.647 | - | 7.374; 7.092 | - |
| **C12** | 7.153 | 4.280 | 3.195; 3.130 | - | - | - |
| **S13** | - | - | - | - | - | - |
| **C14** | 8.648 | 3.933 | 3.401; 2.900 | - | - | - |
| **N15** | 7.961 | 5.023 | 2.752 | - | - | - |
| **T16** | 7.699 | 4.431 | - | 1.261 | - | - |
| **G17** | 8.049 | 4.114 | - | - | - | - |
| **T18** | - | 4.507 | 4.292 | 1.333 | - | - |
| **K19** | 7.771 | 4.515 | 1.823; 1.757 | 1.289 | 1.551 | - |
| **D20** | 8.152 | 4.610 | 2.645; 2.460 | - | - | - |
| **C21** | 8.298 | 5.270 | 3.255; 3.076 | - | - | - |
| **D22** | 8.267 | 4.834 | 2.954; 2.531 | - | - | - |
| **C23** | 8.604 | 4.438 | 3.301; 2.825 | - | - | - |
| **S24** | 9.296 | 4.669 | 4.112; 3.852 | - | - | - |
| **D25** | - | 4.413 | 2.691; 2.647 | - | - | - |
| **A26** | 8.077 | 4.295 | 1.326; | - | - | - |
| **K27** | 8.445 | 4.292 | 1.942 | 1.265 | - | - |
| **C28** | 8.725 | 3.983 | 3.453; 2.878 | - | - | - |
| **C29** | 7.679 | 5.048 | 3.102; 2.792 | - | - | - |
| **E30** | 8.700 | 4.233 | 1.908; 1.802 | 2.343; 2.175 | - | - |
| **Q31** | 8.082 | 4.947 | 2.414; 2.087 | 1.694 | - | 7.624; 8.339 |
| **Y32** | 8.777 | 4.825 | 3.071; 2.585 | - | 6.910 | 6.731 |
| **C33** | 8.166 | 4.273 | 3.269; 2.981 | - | - | - |
| **C34** | 7.530 | 4.901 | 3.444; 3.114 | - | - | - |
| **P35** | - | 4.925 | 2.297; 2.023 | 1.775 | 4.225; 3.749 | - |
| **T36** | 7.933 | 4.504 | 4.066 | 1.336 | - | - |
| **A37** | 8.924 | 4.747 | 1.801 | - | - | - |
| **S38** | 7.337 | 2.375 | 3.794; 3.482 | 6.338 | - | - |
| **E39** | 7.122 | 4.337 | 2.084; | 2.257 | - | - |
| **K40** | 8.474 | 4.068 | 1.979; 1.811 | 1.423 | 1.623 | 3.027 |
| **K41** | 8.773 | 4.109 | 2.104; 2.025 | 1.344; 1.245 | 1.626 | 3.032 |
| **C42** | 7.677 | 4.382 | 2.934; 2.774 | - | - | - |
| **C43** | 7.180 | 4.776 | 3.251; 2.869 | - | - | - |
| **K44** | 8.513 | 4.262 | 1.870; 1.733 | 1.526; 1.461 | 1.688 | 3.007 |
| **S45** | 8.413 | 3.574 | 3.545; 3.446 | - | - | - |
| **G46** | 8.702 | 4.149; 3.412 | - | - | - | - |
| **C47** | 7.221 | 3.998 | 3.229; 2.912 | - | - | - |
| **A48** | 8.581 | 4.374 | 1.449 | - | - | - |
| **G49** | 8.972 | 4.517; 4.235 | - | - | - | - |
| **G50** | - | 4.086; 3.817 | - | - | - | - |
| **C51** | 8.119 | 4.153 | 2.902; 2.770 | - | - | - |
| **K52** | 8.640 | 3.732 | 1.883 | 1.257 | 1.648 | - |
| **C53** | 7.512 | 3.935 | 3.163; 2.765 | - | - | - |
| **A54** | 8.468 | 4.147 | 1.399 | - | - | - |
| **N55** | 9.188 | 4.770 | 2.737; 2.645 | - | 7.267; 7.119 | - |
| **C56** | 7.472 | 5.134 | 3.335; 3.067 | - | - | - |
| **E57** | 8.489 | 4.213 | 2.365; 2.184 | 2.434; 2.541 | - | - |
| **C58** | 7.328 | 4.075 | 3.316; 2.777 | - | - | - |
| **A59** | 7.261 | 3.662 | 1.374 | - | - | - |
| **Q60** | 8.002 | 4.284 | 2.112; 1.929 | 2.334 | - | - |
| **A61** | 8.008 | 4.280 | 1.383 | - | - | - |
| **A62** | 8.212 | 4.284 | 1.346 | - | - | - |
| **H63** | 7.849 | 4.424 | 3.196; 3.059 | - | 7.134 | 8.282 |

**Appendix Table S3.** ^1^H, ^13^C and ^15^N NMR chemical shift data for Zn_7_MTL-1.

| **Residue** | **C(α)** | **N** | **H** | **H(α)** | **H(β)** | **H(γ)** | **H(δ)** | **H(ε)** |
| --- | --- | --- | --- | --- | --- | --- | --- | --- |
| **A1** | - | - | - | - | - | - | - | - |
| **C2** | 57.3 | - | - | - | - | - | - | - |
| **K3** | 56.8 | 131.4 | 9.829 | 4.170 | 1.948 |  | 1.603 | - |
| **C4** | 57.3 | 118.7 | 8.019 | 4.026 | 2.899; 2.645 | - | - | - |
| **D5** | 52.6 | 118.3 | 7.947 | 4.396 | 3.041; 2.335 | - | - | - |
| **C6** | 56.7 | 116.1 | 7.177 | 4.035 | 3.558; 2.886 | - | - | - |
| **K7** | 54.9 | 116.6 | 8.518 | 3.687 | 1.188 | 1.460 | 1.595 | - |
| **N8** | 48.6 | - | - | 4.921 | 3.076 | - | 8.460; 7.352 | - |
| **K9** | 56.3 | 126.4 | 8.884 | 4.031 | 1.990;1.867 | - | - | - |
| **Q10** | 52.6 | 114.6 | 8.109 | 4.360 | 1.896 | 2.286 | - | - |
| **C11** | 59.4 | 124.1 | 7.324 | 4.099 | 3.173; 2.956 | - | - | - |
| **K12** | 55.3 | 128.6 | 8.439 | 4.168 | 1.990; 1.902 | 1.550 | - | - |
| **C13** | 61.7 | 122.9 | 8.413 | 3.900 | 3.059; 2.769 | - | - | - |
| **G14** | 44.1 | 109.5 | 7.974 | 3.672; 3.935 | - | - | - | - |
| **D15** | 53.5 | - | - | - | - | - | - | - |
| **K16** | 52.5 | 120.3 | 7.954 | 4.488 | 1.809 | 1.329; 1.276 | 1.631 | - |
| **C17** | 57.2 | 124.7 | 8.357 | 4.870 | 3.173 | - | - | - |
| **E18** | 52.9 | 132.9 | 9.123 | 4.675 | 1.800 | 2.226 | - | - |
| **C19** | 61.0 | 123.6 | 8.992 | 4.109 | 3.085; 2.843 | - | - | - |
| **S20** | - | - | - | - | - | - | - | - |
| **G21** | - | - | - | - | - | - | - | - |
| **D22** | - | - | - | - | - | - | - | - |
| **K23** | 54.8 | - | - | - | - | - | - | - |
| **C24** | 56.2 | 122.6 | 8.316 | 4.482 | 3.704 | - | - | - |
| **C25** | 54.9 | 120.0 | 7.821 | 4.845 | 3.237; 2.870 | - | - | - |
| **E26** | 54.4 | 121.9 | 8.478 | 4.176 | 1.877 | 2.229 | - | - |
| **K27** | 52.8 | 121.9 | 8.063 | 4.969 | 1.799; 1.435 | 1.035 | 1.560 | 2.961 |
| **Y28** | 55.3 | 119.0 | 8.563 | 4.598 | 2.626; 3.122 | - | 6.848 | 6.701 |
| **C29** | 56.1 | 116.4 | 7.656 | 4.101 | 2.847; 3.251 | - | - | - |
| **C30** | 54.8 | 113.0 | 7.351 | 4.525 | 3.103; 2.924 | - | - | - |
| **E31** | 57.0 | 120.3 | 9.073 | 4.421 | 1.957; 1.733 | 2.179 | - | - |
| **E32** | 58.1 | 123.0 | 8.768 | 4.034 | 2.168; 1.984 | 2.398; 2.347 | - | - |
| **A33** | 54.4 | 126.5 | 8.974 | 4.786 | 1.833 | - | - | - |
| **S34** | 58.3 | 113.7 | 7.393 | 2.204 | 3.826; 3.508 | - | - | - |
| **E35** | 55.7 | 121.3 | 7.208 | 4.354 | 2.023 | 2.230 | - | - |
| **K36** | 54.8 | 117.1 | 8.895 | 4.118 | 2.014; 1.773 | 1.397 | 1.580 | - |
| **K37** | 53.8 | 114.7 | 8.780 | 4.218 | 2.130; 1.988 | 1.175 |  | - |
| **C38** | 55.7 | 116.0 | 7.415 | 4.313 | 2.932; 2.772 | - | - | - |
| **C39** | 54.0 | 119.8 | 7.144 | 4.959 | 3.239; 2.949 | - | - | - |
| **P40** | 60.2 | - | - | 4.490 | 2.232 | 1.992; 1.900 | 3.777; 3.654 | - |
| **A41** | 51.1 | 122.7 | 8.187 | 3.332 | 1.014 | - | - | - |
| **G42** | 42.4 | 112.8 | 9.267 | 4.080; 3.273 | - | - | - | - |
| **C43** | 59.3 | 122.6 | 7.458 | 3.917 | 3.256; 2.828 | - | - | - |
| **K44** | 54.1 | 127.7 | 8.696 | 4.199 | 1.873; 1.661 |  | 1.543 | - |
| **G45** | 44.2 | 110.0 | 9.364 | 4.235; 3.816 | - | - | - | - |
| **D46** | 50.4 | 120.6 | 8.375 | 4.947 | 2.730; 2.511 | - | - | - |
| **C47** | 54.2 | 119.2 | 7.966 | 4.122 | 3.203; 2.782 | - | - | - |
| **K48** | 55.3 | 122.5 | 8.147 | 4.031 | 1.895; 1.851 | 1.343 | - | 2.759 |
| **C49** | 58.3 | 120.0 | 7.318 | 4.027 | 3.321 | - | - | - |
| **A50** | 52.6 | 131.9 | 9.133 | 4.249 | 1.523 | - | - | - |
| **N51** | 53.8 | 117.1 | 9.005 | 4.775 | 2.784; 2.551 |  | 7.570; 7.048 | - |
| **C52** | 57.3 | 117.9 | 8.787 | 4.402 | 3.543; 2.908 | - | - | - |
| **H53** | 52.8 | 113.5 | 7.573 | 4.609 | 3.624; 3.473 | - | 7.139 | 7.470 |
| **C54** | 61.8 | 122.8 | 7.308 | 4.224 | 2.744; 2.689 | - | - | - |
| **A55** | 51.2 | 120.3 | 8.111 | 4.333 | 1.349 | - | - | - |
| **E56** | 55.0 | 118.0 | 9.524 | 3.838 | 2.167 | 2.041 | - | - |
| **Q57** | 55.5 | 107.1 | 7.905 | 3.689 | 2.140 | 2.333 | - | 7.484; 6.706 |
| **K58** | 52.8 | 119.3 | 7.974 | 4.680 | 1.910; 1.803 | 1.474 | - | - |
| **Q59** | 53.3 | 119.2 | 8.167 | 4.814 | 2.144; 1.897 | 2.444 | - | 7.138; 7.700 |
| **C60** | 58.8 | 125.4 | 9.318 | 4.073 | 2.947; 2.645 | - | - | - |
| **G61** | 43.1 | 116.8 | 8.844 | 3.906 | - | - | - | - |
| **D62** | 52.1 | 122.9 | 8.195 | 4.415 | 1.937; 1.895 | - | - | - |
| **K63** | 56.2 | 128.9 | 8.887 | - | - | - | - | - |
| **T64** | 61.1 | 110.9 | 8.871 | 4.715 | 4.345 | 1.220 | - | - |
| **H65** | 52.0 | 120.7 | 7.504 | 4.576 | 3.154; 2.629 | - | 6.752 | 7.823; 6.037 |
| **Q66** |  | - | - | - | - | - | - | - |
| **H67** | 54.4 | - | - | 4.569 | 3.296; 3.051 | - | 7.103 | 7.256 |
| **Q68** | 56.1 | 127.6 | 8.058 | 4.203 | 2.008; 1.894 | 2.244; 2.209 | - | - |
| **G69** | - | - | - | - | - | - | - | - |
| **T70** | - | - | - | - | - | - | - | - |
| **A71** | - | - | - | - | - | - | - | - |
| **A72** | - | - | - | - | - | - | - | - |
| **A73** | 50.3 | - | - | - | -  - | - | - | - |
| **H74** | 55.3 | 123.8 | 7.725 | 4.373 | 3.104; 2.982 | - | 6.950 | 7.861 |

**Appendix Table S4.** Pdb entries harbouring His_3_Cys sites. With the exception of MTL-1, entries and related information were initially retrieved from Metal-pdb (Putignano et al., 2018) and reviewed. The first two entries are representatives for a total of 19 related entries with equistructural sites, all pertaining to bacterial or archaeal tRNA synthetases. The location and arrangement of coordinating residues in MTL-1 is unique.

| **PDB Chain** | **Molecule Name** | **Organism Name** | **UniProt Id** | **Sequence length** | **Zn-coordinating residues** |
| --- | --- | --- | --- | --- | --- |
| 8aq9 | MTL-1 | *C. elegans* | P17511 | 75 | H53,C60,H65,H67 |
| 1nyr_3 | Threonyl-tRNA synthetase | *Staphylococcus aureus* | Q8NW68 | 645 | H75,H79,C181,H185 |
| 1v4p_C | Alanyl-tRNA editing protein AlaX-S | *Pyrococcus horikoshii* | [O58307](http://www.uniprot.org/uniprot/O58307) | 157 | H9,H13,C116,H120 |
| 2l0z_A | Pre-glycoprotein polyprotein GP complex | *Junin virus* | [P26313](http://www.uniprot.org/uniprot/P26313) | 485 | H447,H449,C455,H485 |
| 3mo0 (A and B) | Histone-lysine N-methyltransferase EHMT1  (SET/pre-SET domains) | *Homo sapiens* | [Q9H9B1](http://www.uniprot.org/uniprot/Q9H9B1) | 1298 | C1014(A),H1027(B), H1076(B),H1185(B) |
| 3t9o_A | Diguanylate cyclase YdeH (DgcZ) | *Escherichia coli K-12* | [P31129](http://www.uniprot.org/uniprot/P31129) | 296 | H22,C52,H79,H83 |
| 5uam_A | Ulvan lyase-PL25 | *Pseudoaltero-monas sp. PLSV* | A0A1W2VMZ5 | 489 | H208,H264,C266,H278 |

Putignano V., Rosato A., Banci L., Andreini C. (2018). **MetalPDB in 2018**: a database of metal sites in biological macromolecular structures. *Nucleic Acids Res.* 46(D1):D459-D464.

**Appendix Table S5**. Statistical analysis of lifespan data. The lifespan was assessed in in WT, *mtl-1*(tm1770) and *mtl-2*(gk125) raised either on control plates or plates supplemented with 150 µM Zn or 30 µM Cd. The statistical analysis was performed using the Logrank (Mantel- Cox) Test for comparison of survival curves (n=400 per condition), * p≤0.05 and **** p≤0.0001.

| Median Survival (days) | | | |
| --- | --- | --- | --- |
|  | WT | *mtl-1*(tm1770) | *mtl-2*(gk125) |
| Control | 14 | 11 | 16 |
| 150 µM Zn | 14 | 13 | 17 |
| 30 µM Cd | 12 | 11 | 10 |

| Statistical analysis: exposures (compared to unexposed) | | |  |
| --- | --- | --- | --- |
|  | 150 µM Zn | 30 µM Cd | |
| WT |  |  | |
| *mtl-1*(tm1770) | **** |  | |
| *mtl-2*(gk125) |  | **** | |

**Appendix Table S6. Structural statistics for Cd_6_MTL-2**

|  | No Cd-S restraints | | With Cd-S restraints | |
| --- | --- | --- | --- | --- |
| Experimental data |  | | | |
| Number of NOE-derived restraints | 477 | | | |
| Number of Cd-S assignments (domain 2 only)  Additionally inferred Cd-S restraints (domain 2 only) | | | 5  7 | |
|  |  | |  | |
| Structural statistics; 20 best conformers |  | |  | |
| CYANA target function (Å) | 2.41±0.46 | | 2.93±0.88 | |
| Violated restraints (>0.25 Å) | 0 | | 0 | |
| Structure validation (model 1) |  | |  | |
| Ramachandran plot analysis (Procheck)  most favoured regions  allowed  disallowed | 26.0%  71.5%  3.5% ^a)^ | | 29.8%  68.4%  1.8% ^a)^ | |
|  |  | |  | |
| RMSD (backbone heavy atoms) | 3.49±0.72 | | 2.62±0.80 | |
|  | domain 1 (3-30) | domain 2 (31-61) | domain 1 (3-30) | domain 2 (31-61) |
|  | 3.26±1.02 | 1.47±0.72 | 2.54±1.05 | 1.18±0.26 |

a) These outliers are located in domain 1 (includes Val2)

**Appendix Table S7. Structural statistics for domain 2 of Zn_7_MTL-1 (residues 26-69).**

| Experimental data |  | | |
| --- | --- | --- | --- |
| Total number of NOE-derived restraints | 314 | | |
| Angle restraints from HNHA and HNHB  Inferred restraints (site G) | 65  4 | | |
|  | without inferred restraints | with inferred restraints |  |
| Structural statistics; 20 best conformers |  |  |  |
| CYANA target function | 1.09±0.18 | 1.73±0.18 |  |
| Violated restraints (>0.25Å) | 0 | 0 |  |
| RMSD (backbone heavy atoms) | 1.94±0.57 | 1.46±0.30 |  |
| Structure validation (model 1) |  |  |  |
| Ramachandran plot analysis (ProCheck)  most favoured regions  allowed  disallowed | 36.8%  63.2%  0.0% | 42.1%  57.9%  0.0% |  |

**Appendix Table S8.** Theoretical and experimental masses for preparations for “tail-swap” mutants. All masses reported are for the singly-charges species

| Preparation | Experimental mass (major species) (Da) | Theoretical mass (Da) |
| --- | --- | --- |
| apo-MTL-1Δ57-71 | 6309.9 | 6311.3 (-Met) ^a)^ |
| Zn-MTL-1Δ57-71 | 6690.7 | 6691.5 (-Met+6Zn^2+^) |
| Cd-MTL-1Δ57-71 | 6973.3 | 6973.8 (-Met+6Cd^2+^) ^b)^ |
|  |  |  |
| apo-MTL-2+MTL-1_57-71 | 8118.9 | 8120.3 (-Met) |
| Zn-MTL-2+MTL-1_57-71 | 8562.3 | 8563.7 (-Met+7Zn^2+^) |
| Cd-MTL-2+MTL-1_57-71 | 8845.1 | 8845.8 (-Met+6Cd^2+^+Zn^2+^) |
|  | 8955.2 | 8955.2 (-Met+7Cd^2+^+Zn^2+^)^c)^ |

^a)^ “-Met” refers to species with the N-terminal Met cleaved; “+Met” to species where this Met is still present.

^b)^ A minor species including the N-terminal Met was also present (7104.25)

^c)^ Other species observed include -Met+6Cd^2+^ (8782.1), -Met+7Cd^2+^ (8892.3), +Met+7Cd^2+^ (9023.15), +Met+7Cd^2+^+Zn^2+^ (9086.4), +Met+8Cd^2+^ (9133.6)

**Appendix Figure S1.** A transgenic nematode overexpressing MTL-1. In detail, the *mtl-1* promoter was cloned into pPD95.75, in frame, to express MTL-1 with the native stop codon. The construct was injected with P*myo-4*::GFP as co-injection marker into the gonads of L4 worms. The next generation was scored for successful transgenics by screening for GFP signal, then sorted to identify worms displaying high penetrance of transfer to subsequent generations. The purpose of this experiment was to explore whether the availability of additional MTL-1 would amplify the accumulation of Zn. Note the Zn hotspot in the anterior part of the gut (asterisk) as well as the anus (arrow).


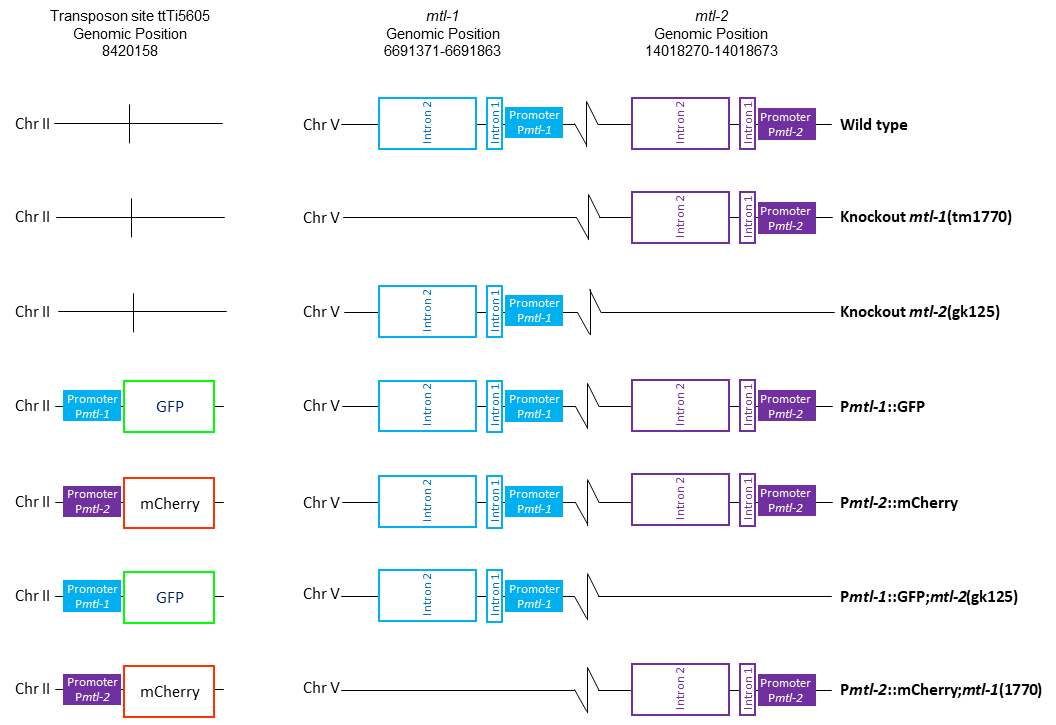


**Appendix Figure S2.** Schematic diagrams of strains used in this study. Wild type and *mtl-2*(gk125) were sourced from the Caenorhabditis Genetics Centre (CGC), *mtl-1*(tm1770) was generated by the National BioResource Project (NBRP).


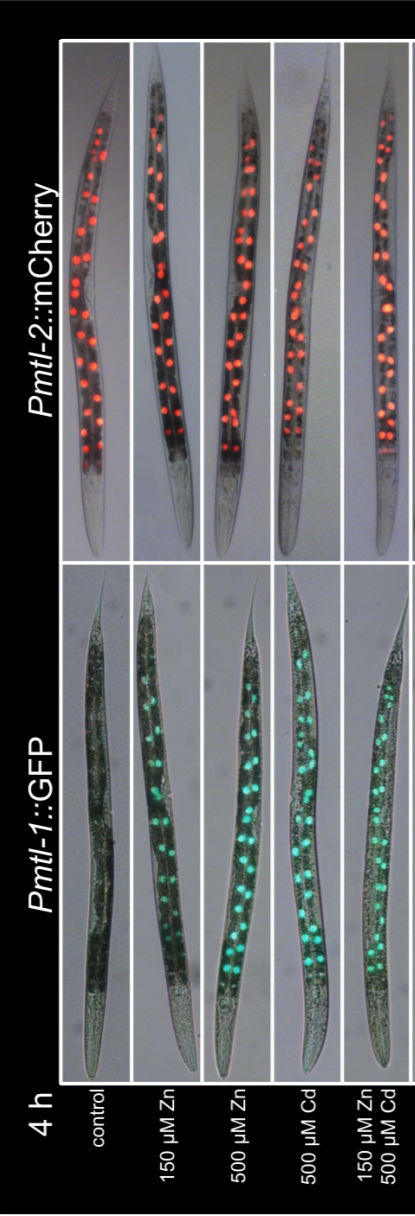

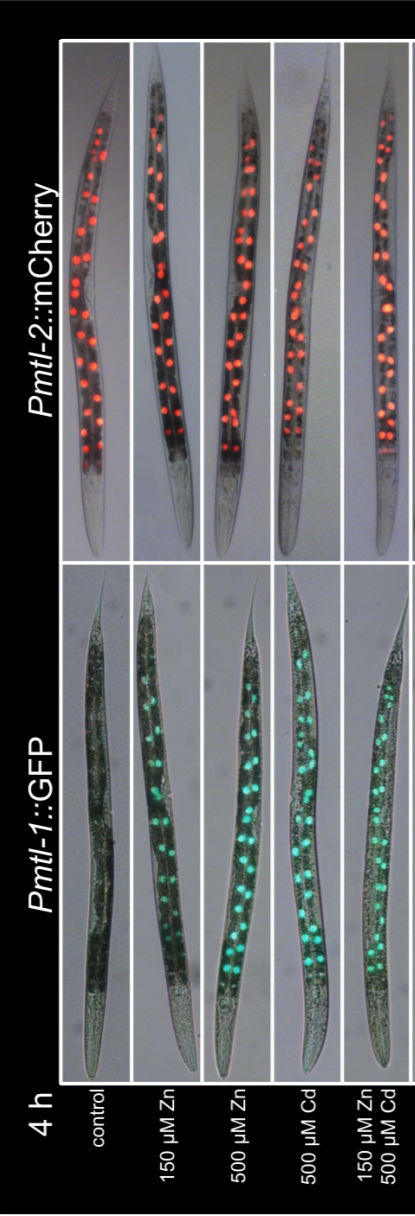

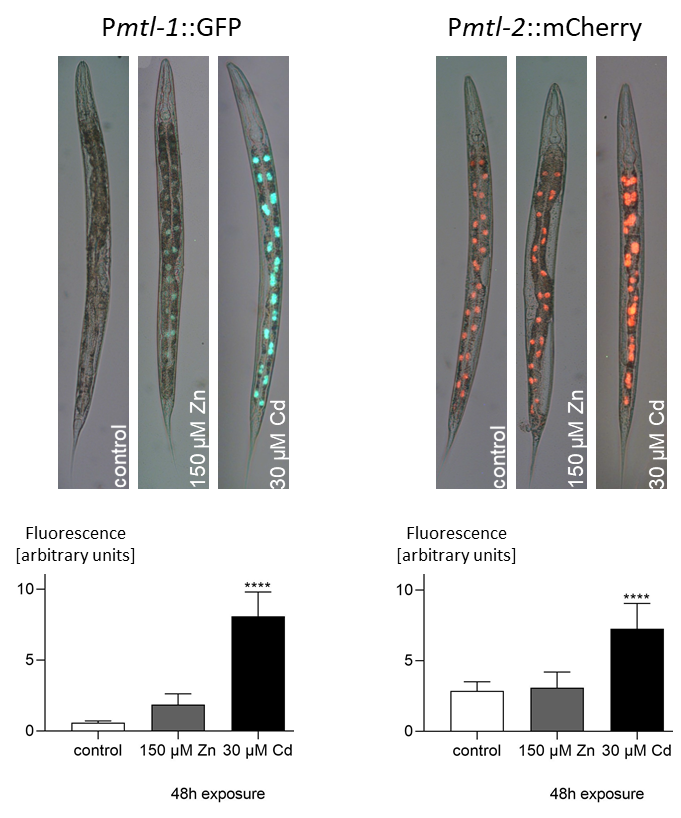

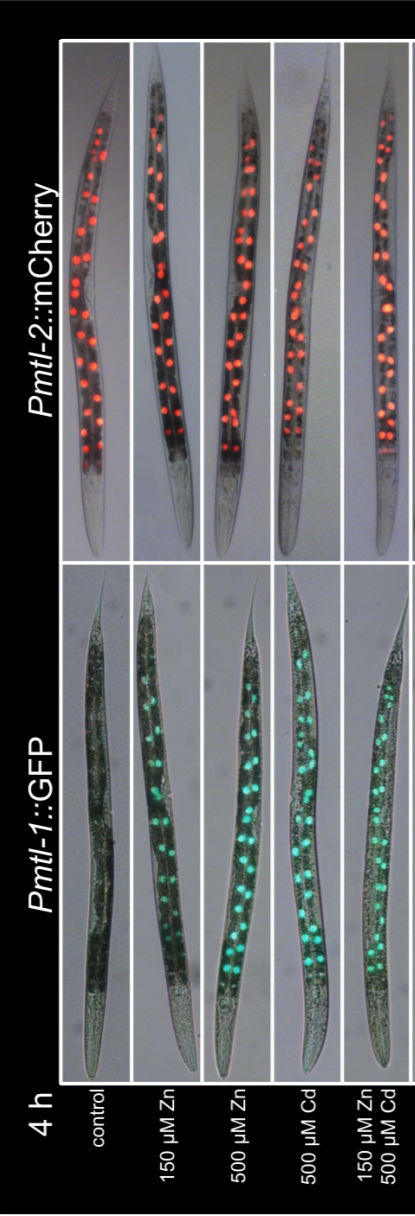

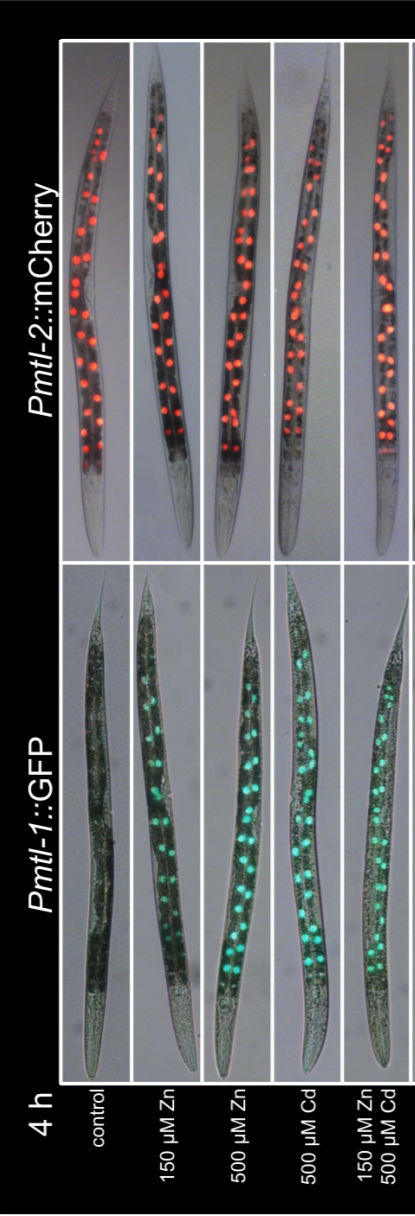

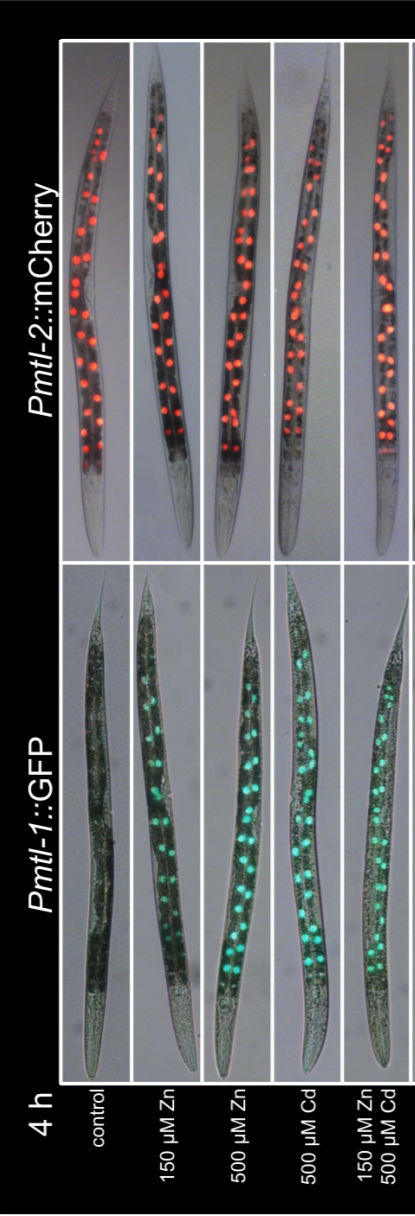

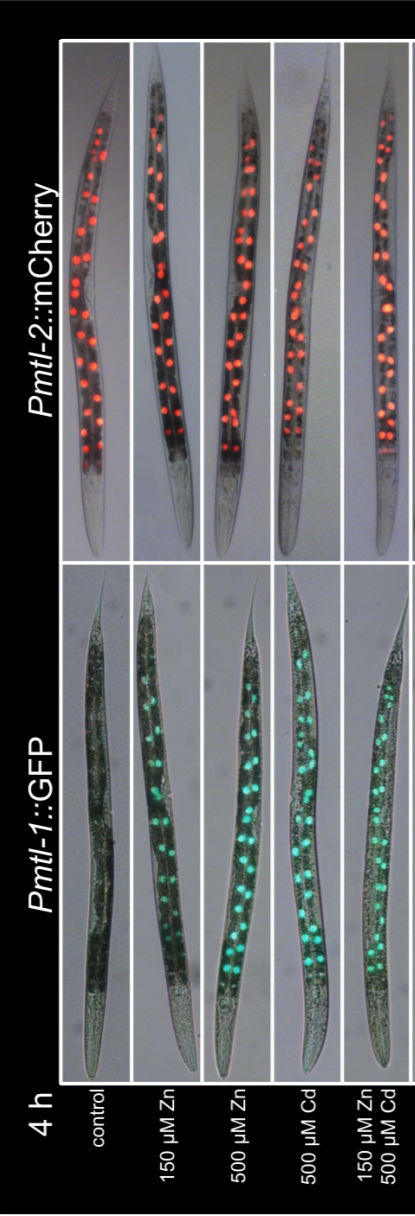

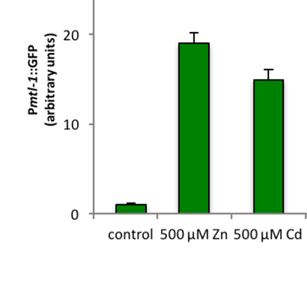

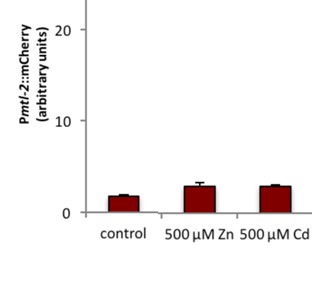


**4h exposure**

**4h exposure**

**A**

**B**

**Appendix Figure S3.** Nematodes (P*mtl-1*::GFP or P*mtl-2*::mCherry) were first grown on standard NGM agar plates for 44 h and then transferred to either unexposed plates or plates supplemented with 500 µM Zn or 500 µM Cd for a further 4 h. All nematodes were age synchronised and analysed at L4 stage and images were captured with a Nikon camera (Nikon UK Ltd., Kingston upon Thames, UK) at 20 × magnification by using blue laser scanning fluorescence (λex = 450 – 490 nm) to excite the GFP and green laser scanning fluorescence (λex = 575 – 620 nm) to excite the mCherry **(A)**. and analysed at L4 stage. For quantitative analysis of fluorescence, 10 transgenic nematodes were examined per condition **(B)**.

**Appendix Figure S4.** Fingerprint regions of 1D ^1^H NMR spectra for different preparations of MTL-1 and MTL-2.

**Appendix Figure S5.** Summary of NOE-derived distance restraints (a and b) and ensemble of 20 best conformers for Cd_6_MTL-2 using ^1^H NMR data only (c).

**Appendix Figure S6**. Multiple sequence alignment of MTs from *Caenorhabditis* species. Sequences were retrieved from Wormbase (<https://wormbase.org/>) and aligned using MUSCLE.

**Appendix Figure S7.**  Observed non-sequential NOE interactions involving the aromatic protons of Tyr32 in Cd_6_MTL-2.

**Appendix Figure S8.** pH titration of Zn_7_MTL-1 monitored by ^1^H NMR (500 MHz; 1 mM protein, 50 mM Tris-d_11_, 50 mM NaCl, 100% D_2_O, 303 K). (a) 1D ^1^H NMR spectra at different pH values. (b) Plot of titration curves for the Hε1 protons. For each imidazole sidechain, two protons, Hε1 and Hδ2, are observed. His74 behaves like a free histidine, showing large changes in chemical shifts around pH 7 (as indicated by the red lines) which are due to protonation. In contrast, the chemical shifts for the other three histidine residues remain constant down to pH 4.88 (as indicated by the blue line for His65 as example), consistent with binding Zn^2+^ as suggested also by ^15^N NMR chemical shifts for the imidazole nitrogens (Figure 5b), and structure determination without inclusion of metal-ligand restraints (Supplementary Figure S9). However, the intensities of the original signals decrease, which is an indication for exchange broadening, particularly evident in the spectrum at pH 4.88. Below this pH, sharp signals for the His protons appear at ca. 8.6 and 7.3 ppm, indicative of protonation and loss of structure. (c) ESI-MS suggests that around this pH, a Zn_6_MTL-1 form dominates speciation.

**Appendix Figure S9.** Summary of NOE-derived distance restraints (a and b) and ensembles of 20 best conformers for domain 2 of Zn_7_MTL-1 using (c) NOE and HNHA data only and (d) including inferred metal-ligand restraints for site G.

**Appendix Figure S10.** Overlay of representative structures (model 1 out of 20 conformers) for the monomeric site G between the final refinement including metal-ligand and ligand-ligand restraints (CPK colouring) with refinement using NOE and HNHA coupling constants only (black). This demonstrates that the ^15^N and ^1^H-NMR based restraints are sufficient to define this site and justify the subsequent incorporation of metal-ligand and ligand-ligand restraints in the final refinement.

**Appendix Figure S11**. Comparison of backbone angles between domains 2 of MTL-1 (green) and MTL-2 (red). Model 1 from the final Zn-MTL-1 ensemble and model 3 from the final Cd-MTL-2 (i.e. those also shown in Figures 4 and 5) were used for this comparison. Especially psi angles differ significantly between the two structures.

**Appendix Figure S12.** Comparison of chemical shifts for conserved residues in domains 2 of Zn_7_MTL-1 and Cd_6_MTL-2. The differences incorporate both NH and CH(α) shifts; Δδ ppm =$\sqrt{{\Delta\delta ppm\left( NH \right)}^{2}+{\Delta\delta ppm(CH\left( \alpha\right))}^{2}}$.

**Appendix Figure S13.** Fingerprint regions of 1D ^1^H NMR spectra for different preparations of the “tail-swap” mutants.

**Appendix Figure S14**. Chemical shift perturbation resulting from deletion of the C-terminal extension for Zn-loaded MTL-1. The differences incorporate both NH and CH(α) shifts; Δδ ppm =$\sqrt{{\Delta\delta ppm\left( NH \right)}^{2}+{\Delta\delta ppm(CH\left( \alpha\right))}^{2}}$.


 **Appendix Figure S15.** (a) Fingerprint region of a 2D TOCSY spectrum for Cd-MTL-1Δ57-71. A selection of residues that were assignable in the Cd-loaded form but not in the Zn-loaded form are highlighted. (b) Chemical shift comparison between Cd-MTL-1Δ57-71 and wild-type Zn-MTL-1. The differences incorporate both NH and CH(α) shifts; Δδ ppm =$\sqrt{{\Delta\delta ppm\left( NH \right)}^{2}+{\Delta\delta ppm(CH\left( \alpha\right))}^{2}}$


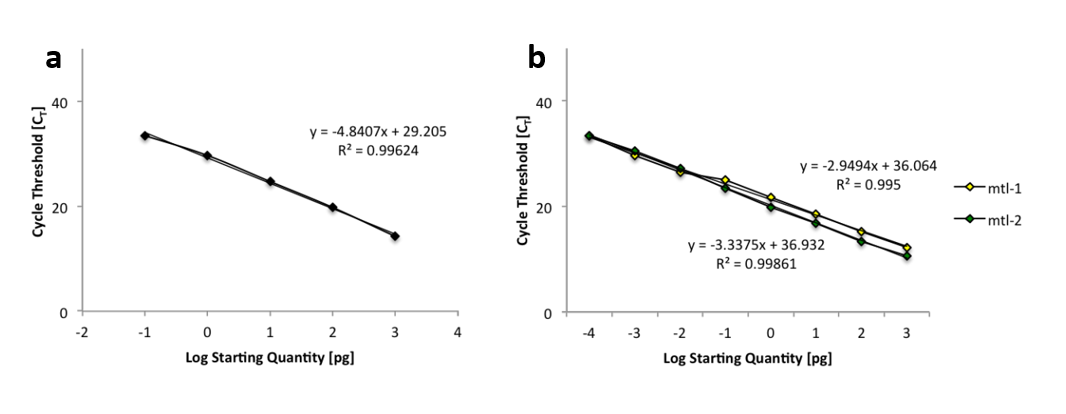


**Appendix Figure S16.** Standard curve of cloned *rla-1* (a) and *mtl-1*, *mtl-2* (b). The CT-value is plotted against the initial plasmid concentration. All lines show an R-squared value of > 0.99, which indicates a good fit of the regression line. The linear equation was used to concert the CT-value of any sample to the initial concentration of the gene, which was transformed to calculate the copy numbers of *rla-1*, *mtl-1* and *mtl-2*, respectively.
