# Supplementary material for: Juggling cadmium detoxification and zinc homeostasis: A division of labour between the two C. elegans metallothioneins
Source: Chemosphere. 2024 Feb;350:141021. doi: 10.1016/j.chemosphere.2023.141021 (PMC11134313; doi:10.1016/j.chemosphere.2023.141021)
Supplement: Multimedia component 2 [file mmc2.docx]

| *mtl* copy number  (normalized) | wild type | | | *mtl-1*(tm1770) | | *mtl-2*(gk125) | |
| --- | --- | --- | --- | --- | --- | --- | --- |
|  | ***mtl-1*** | ***mtl-2*** | **Total *mtl*** | ***mtl-1*** | ***mtl-2***  **(total *mtl*)** | ***mtl-1***  **(total *mtl*)** | ***mtl-2*** |
| no metal | 1 | 86 | 87 | 0 | 58 | 7 | 0 |
| Zn (150 µM) | 5 | 339 | 343 | 0 | 79 | 9 | 0 |
| Cd (30 µM) | 45 | 1356 | 1401 | 0 | 813 | 132 | 0 |

| net *mtl* compared to wild type | *mtl-1* | | *mtl-2* | | Total *mtl* | |
| --- | --- | --- | --- | --- | --- | --- |
|  | ***mtl-1***  **(tm1770)** | ***mtl-2* (gk125)** | ***mtl-1***  **(tm1770)** | ***mtl-2* (gk125)** | ***mtl-1***  **(tm1770)** | ***mtl-2* (gk125)** |
| no metal | 0 | +7 | -28 | -86 | -29 | -80 |
| Zn (150 µM) | -5 | +4 | -260 | -339 | -264 | -334 |
| Cd (30 µM) | -45 | +87 | -543 | -1356 | -588 | -1269 |
